# Variation in mitogenome structural conformation in wild and cultivated lineages of sorghum corresponds with domestication history and plastome evolution

**DOI:** 10.1186/s12870-023-04104-2

**Published:** 2023-02-13

**Authors:** Shuo Zhang, Jie Wang, Wenchuang He, Shenglong Kan, Xuezhu Liao, David R. Jordan, Emma S. Mace, Yongfu Tao, Alan W. Cruickshank, Robert Klein, Daojun Yuan, Luke R. Tembrock, Zhiqiang Wu

**Affiliations:** 1 National Key Laboratory of Crop Genetic Improvement, Huazhong Agricultural University, Hubei Wuhan, 430070, China; 2 Shenzhen Branch, Guangdong Laboratory for Lingnan Modern Agriculture, Genome Analysis Laboratory of the Ministry of Agriculture, Agricultural Genomics Institute at Shenzhen, Chinese Academy of Agricultural Sciences, Guangdong Shenzhen, 518120, China; 3 Queensland Alliance for Agriculture and Food Innovation (QAAFI), Hermitage Research Facility, The University of Queensland, Warwick, Queensland 4370, Australia; 4 Department of Agriculture and Fisheries (DAF), Agri-Science Queensland, Hermitage Research Facility, Warwick, Queensland 4370, Australia; 5 Southern Plains Agricultural Research Center, USDA-ARS, College Station, Texas 77845, USA; 6 Department of Agricultural Biology, Colorado State University, Fort Collins, Colorado 80523, USA

**Keywords:** Mitochondrion, Chloroplast, Intracellular transfer, Crop improvement, Grains

## Abstract

**Background:**

Mitochondria are organelles within eukaryotic cells that are central to the metabolic processes of cellular respiration and ATP production. However, the evolution of mitochondrial genomes (mitogenomes) in plants is virtually unknown compared to animal mitogenomes or plant plastids, due to complex structural variation and long stretches of repetitive DNA making accurate genome assembly more challenging. Comparing the structural and sequence differences of organellar genomes within and between sorghum species is an essential step in understanding evolutionary processes such as organellar sequence transfer to the nuclear genome as well as improving agronomic traits in sorghum related to cellular metabolism.

**Results:**

Here, we assembled seven sorghum mitochondrial and plastid genomes and resolved reticulated mitogenome structures with multilinked relationships that could be grouped into three structural conformations that differ in the content of repeats and genes by contig. The grouping of these mitogenome structural types reflects the two domestication events for sorghum in east and west Africa.

**Conclusions:**

We report seven mitogenomes of sorghum from different cultivars and wild sources. The assembly method used here will be helpful in resolving complex genomic structures in other plant species. Our findings give new insights into the structure of sorghum mitogenomes that provides an important foundation for future research into the improvement of sorghum traits related to cellular respiration, cytonuclear incompatibly, and disease resistance.

**Supplementary Information:**

The online version contains supplementary material available at 10.1186/s12870-023-04104-2.

## Background

Mitochondria and plastid in plant cells are thought to have evolved from α-proteobacteria and cyanobacteria subsumed by eukaryotic ancestors in at least two endosymbiosis events and made progressively less autonomous through gene transfer with the host cell nucleus [1–4]. Like other aerobic eukaryotes, plant mitochondria contain a genome (mitogenome) that encodes important components of the electron transport chain (ETC) and controls the expression of these genes [5–7]. But unlike animals in which many mitogenomes have been assembled and annotated, fewer complete plant mitogenomes are available, limiting our understanding of plant cellular evolution [8]. Moreover, unlike most animals, plant mitogenomes can vary greatly in size and structure as seen in the mitogenomes of *Silene latifolia* at 0.25 Mb and *Larix sibirica* at 11.7 Mb [8–11]. Additionally, the master circle model applicable to most animal mitogenomes does not explain the structure found in many plant species with linear, branched chain, or multiple circular architectures resolved from numerous lineages [12–14]. Such structural complexity underlies the lack of completed mitogenomes available in public repositories. The persistence of mitochondria and plastids in plant cells has resulted in a codependent asymmetric pattern where organellar DNA is targeted to the nucleus while gene products are targeted to the organelles from the nucleus [15]. The evidence of ongoing bombardment of the nucleus by organellar DNA has been found in the many nuclear mitochondrial DNAs (NUMTs) and nuclear plastid DNAs (NUPTs) [16]. Although most organelle-derived nuclear DNA is considered inactive, there is evidence that some of the transferred DNA is transcribed in plant species from studies in rice and cotton [17]. Such transferred fragments are expected to affect nuclear genome evolution in a number of ways including possible infertility through cytonuclear incompatibility [18]. Moreover, studies of mitogenome structure in plants are mainly conducted in model species, such as *Arabidopsis thaliana*, *Oryza sativa* (rice), and *Zea mays* (maize), limiting the application of generalizable inferences about the evolution and function for plant mitogenomes. As such a greater diversity of plant lineages, including within species comparisons, should be studied to improve our understanding of plant mitogenomes.

Sorghum (*Sorghum bicolor*) is the fifth most important cereal crop worldwide in terms of production and area planted [19]. Sorghum has attracted much attention in recent years as a crop with great potential to address global food security challenges, because it is a C4 species well adapted to semi-arid and arid environments [19, 20]. Recently, genome research on sorghum has yielded a reference genome [21], the establishment of mutant libraries, gene expression maps, identification of many of the key loci and genes controlling agronomic and adaptive traits, and a pangenome resolved from 13 diverse accessions of *Sorghum* [22–32]. However, much of this genomic data has yet to be applied to rigorous breeding and gene editing projects in the development of improved sorghum cultivars. An important gap in the genomic knowledge for sorghum appears in the limited number of studies related to organelle evolution, structure, and function, especially as pertains to agronomic trait improvement. The study of plant organellar genomes is applicable to numerous agronomic traits. For example, abnormal development of chloroplasts has been shown to result in incomplete development of grains, resulting in reduced yield and quality [33], while mitochondria are known to be an integral part of disease and cold resistance [34, 35]. In sorghum the mitogenome is known to contribute to the widely used A1 cytoplasmic male sterility system, which has great economic impact [36–39]. Despite the importance of organelles in plant survival and improvement of agronomic traits, mitogenome assembly and comparison of multiple sorghum individuals has yet to be completed.

Within the *Sorghum* genus few organellar genome resources are publicly available to study the above evolutionary phenomena with only four plastid genomes (plastomes) [40, 41] and one simplified master circle mitogenome, assembled from short read next generation sequencing data (NCBI accession number: NC_008360.1). To improve genomic studies in the important staple crop sorghum, the mitogenomes and plastomes from six *S. bicolor* accessions, and one closely related species, *S. propinquum*, were assembled from long-read sequence data. Unlike most of the published simplified master circle mitochondrial structures, reticulate mitochondrial conformations with multiple junctions were resolved here, with junctions further verified with PCR experiments. With the assembled organellar genomes, comparisons between domesticated and wild sorghum were made to assess whether any loci or structural arrangements could be associated with domestication. Furthermore, we quantified and characterized organellar sequence transfer to the nucleus between the different sorghum accessions. This study not only improves our understanding of sorghum organellar genome structure and evolution but also provides a basis for the improvement of cultivars through selection of organelles associated with important agronomic traits such as cold and disease resistance, management of cytoplasmic male sterility, and improved yield.

## Results

### Sorghum organellar genomes

**Fig. 1:**
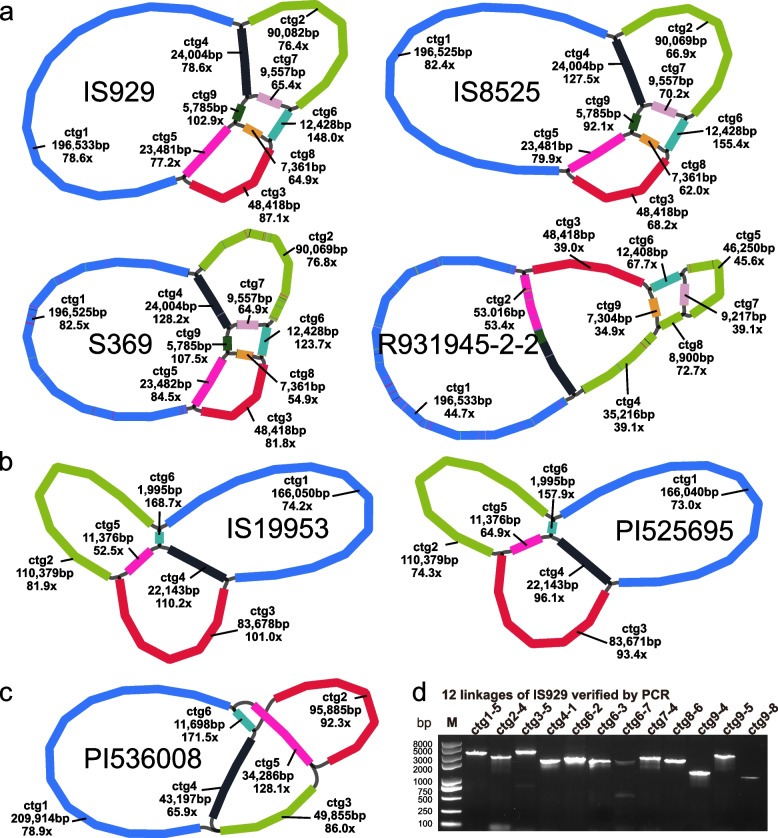
Mitogenome structure from seven sorghum accessions generated using Bandage. **a **type I genomes with 9 contigs from accession IS929, IS8525, S369 and R931945-2–2, among which R931945-2–2 possessed two contigs with differences in linkage. The nine contigs of IS929 were mapped to all the type I genomes, and the coloring indicates their correspondence in each genome. **b** type II genomes with six contigs from IS19953 and PI525695. The coloring of the contigs indicates the correspondence of the six contigs of IS19953 with those in PI525695. **c** type III genome with six contigs, from PI536008. **d** PCR amplification to verify all 12 linkages of the type I conformation in IS929. The numbers above each lane of the gel refer to linkages spanned by the primers in respect to the contig

To obtain a more comprehensive understanding of the mitogenome of sorghum, results from different sequencing (short Illumina reads and long PacBio CLR reads) and assembly methods were combined to assemble the mitogenomes of seven sorghum accessions. The total size of these seven mitogenomes ranged from 395,604 bp to 444,835 bp, which is similar to most other land plant mitogenomes assembled thus far. The average read coverage for each accession was ~ 83x (Table 1), except for R931945-2–2 which was only 45.8x (Fig. 1a). The mitogenomes were divided into three types based on structure which were 417 kbp (Type I), 395 kbp (Type II), and 444 kbp (Type III) in length. Type I included accessions IS929, IS8525, R931945-2–2, S369, type II IS19953 and PI525695, and type III PI536008 (Fig. 1a-c). The GC content of the seven mitogenomes varied from 43.69 to 43.73%, which is also similar to the previously published mitogenome for sorghum at 43.7% (Table 1). Although there were differences in contig size and number among the three mitogenome types, in all genomes 32 protein-coding genes (PCGs) were annotated, which included complex I (NADH Dehydrogenase Subunits, *nad1*, *nad2*, *nad3*, *nad4*, *nad4L*, *nad5*, *nad6*, *nad7*, *nad9*), complex III (Cytochrome bc1 Complex Subunits, *cob*), Complex IV (Cytochrome c Oxidase Subunits, *cox1*, *cox2* and *cox3*), Complex V (ATP Synthase Subunits, *atp1*, *atp4*, *atp6*, *atp8*, and *atp9*), cytochrome c maturation proteins (*ccmB*, *ccmC*, *ccmFc*, and *ccmFn*), ribosomal proteins (*rps1*, *rps2*, *rps3*, *rps4*, *rps7*, *rps12*, *rps13*, and *rpl16*), and other proteins (*mttB*, and *matR*). Among the protein coding genes (PCGs), eight genes contained introns, three of which (*ccmFC*, *cox2*, and *rps3*) contained one intron, and five others (*nad1*, *nad2*, *nad4*, *nad5*, and *nad7*) contained two or more introns. The exons of *nad1*, *nad2*, and *nad5* either occurred at distant positions in a long contig or were split between multiple contigs.

**Table1:** Iterative assembly methods for seven mitogenomes of sorghum

Accession	Lineage	Cultivar	Origin	Raw CLR data/Gbp	Mapped CLR data/Mbp	Self-Corrected CLR data/Mbp	Assembler	Contigs number	Length of Contigs/bp	Length of Polished Contigs/bp	GC%
IS929	*S. bicolor* ssp.* bicolor*	Durra	Sudan	10	238	42	Flye-meta	9	416,821	None	43.71
IS8525	*S. bicolor* ssp.* bicolor*	Kafir	Ethiopia	10	209	35	Flye-meta	8	440,024	None	43.79
R931945-2–2	*S. bicolor* ssp.* bicolor*	Complex	Australia	10	118	26	Flye-meta	9	415,573	None	43.7
S369	*S. propinquum*	Wild relative	Philippines	10	134	36	Flye-meta	8	439,979	None	43.79
IS19953	*S. bicolor* ssp.* bicolor*	Margaritiferum	Sierra Leone	10	285	43	Flye-meta	8	394,502	None	43.72
PI525695	*S. bicolor* ssp.* bicolor*	Margaritiferum	Mali	10	249	42	Flye-meta	2	434,849	None	43.85
PI536008	*S. bicolor* ssp. *verticilliflorum*	Wild progenitor	Cameroon	10	177	38	Flye-meta	6	463,226	None	43.82
IS929	*S. bicolor* ssp.* bicolor*	Durra	Sudan	41	402	47	Flye-meta	6	440,022	None	43.79
IS8525	*S. bicolor* ssp.* bicolor*	Kafir	Ethiopia	55	395	40	Flye-meta	5	440,024	None	43.79
R931945-2–2	*S. bicolor* ssp.* bicolor*	Complex	Australia	10	118	26	Flye-meta	9	415,573	None	43.7
S369	*S. propinquum*	Wild relative	Philippines	26	227	49	Flye-meta	5	449,184	None	43.71
IS19953	*S. bicolor* ssp.* bicolor*	Margaritiferum	Sierra Leone	24	407	50	Flye-meta	6	394,513	None	43.72
PI525695	*S. bicolor* ssp.* bicolor*	Margaritiferum	Mali	24	368	44	Flye-meta	2	434,847	None	43.85
PI536008	*S. bicolor* ssp. *verticilliflorum*	Wild progenitor	Cameroon	40	343	44	Flye-meta	6	443,706	None	43.71
IS929	*S. bicolor* ssp.* bicolor*	Durra	Sudan	41	402	47	SPAdes	9	417,606	417,669	43.71
IS8525	*S. bicolor* ssp.* bicolor*	Kafir	Ethiopia	55	395	40	SPAdes	9	417,544	417,648	43.71
R931945-2–2	*S. bicolor* ssp.* bicolor*	Complex	Australia	10	118	26	SPAdes	9	417,178	417,262	43.7
S369	*S. propinquum*	Wild relative	Philippines	26	227	49	SPAdes	9	417,603	417,648	43.71
IS19953	*S. bicolor* ssp.* bicolor*	Margaritiferum	Sierra Leone	24	407	50	SPAdes	6	395,555	395,621	43.72
PI525695	*S. bicolor* ssp.* bicolor*	Margaritiferum	Mali	24	368	44	SPAdes	6	395,556	395,604	43.73
PI536008	*S. bicolor* ssp. *verticilliflorum*	Wild progenitor	Cameroon	40	343	44	SPAdes	6	444,770	444,835	43.69

**Fig. 2:**
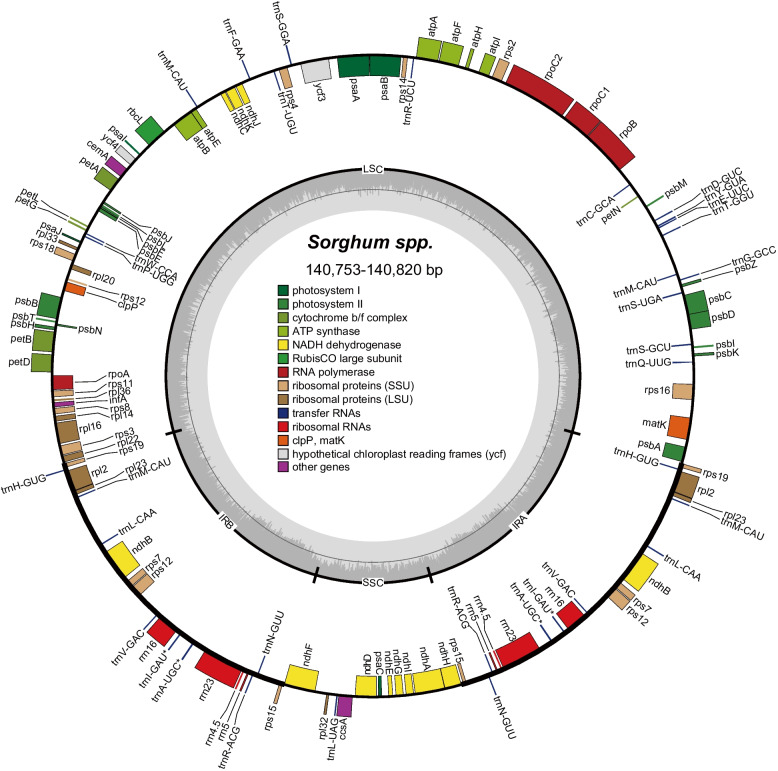
Plastome structure of sorghum. Genes mapped outside the outer circle are transcribed counterclockwise, and those inside are transcribed clockwise. Genes are color coded by functional group. The LSC (large single copy region), SSC (small single copy region), and the IRA and IRB (inverted repeats) are indicated on the inner circle along with GC content in dark gray and AT content in lighter gray

The plastome size across the seven sorghum accessions was very similar, varying from 140,753 to 140,820 bp (Fig. 2). Using plastome size the accessions can be categorized into two groups containing IS929 (140,753 bp), IS8525 (140,753 bp), R931945-2–2 (140,754 bp) and S369 (140,753 bp) with the second group containing IS19953 (140,820 bp), PI525695 (140,819 bp), and PI536008 (140,820 bp) (Fig. 2; Suppl. Table 1). This grouping of plastome sizes mirrors the grouping of mitogenomes by contig number. All of the assembled plastomes contained 104 unique genes, including 76 protein coding genes, 24 tRNA, and four rRNA, with the IR region containing seven protein coding genes, 8 tRNA and four rRNA, with 10 PCGs containing introns, two of these (*rps12* and *ycf3*) have more than one intron. The GC content of the assembled plastomes varied from 38.7–38.9% (Fig. 2; Suppl. Table 1). Overall, the length of the plastomes has diverged among the different subspecies of sorghum, as with the mitogenomes.

### Diversity of mitogenome structure and sequence in sorghum

To determine the variability in structure between different sorghum mitogenomes, we employed a novel strategy. First, we used next-generation sequencing data to de novo assemble the mitogenome, filtering mitochondrial sequences as ‘baits’ to extract CLR long reads. The long reads were assembled using two methods after self-correction, the first method employed the software Flye which uses a repeat graph assembly algorithm for assembling long-read data and can provide unique and stable contigs. The second software SPAdes uses a DeBruijn-graph (DBG) assembly algorithm to break long reads into segments of different lengths according to differences in k-mer values, a connection network of short contigs are assembled according to the overlapping relationship between k-mers. Lastly, stable contigs obtained by Flye were mapped to the connection network obtained by SPAdes to determine the final genome structure. Sorghum mitogenome structure was obtained after three iterations of assembly (Table 1). In the first assembly iteration, the mitogenome conformation varied greatly in different accessions, however a high degree of contig consistency was found among IS929, IS8525, R931945-2–2 and S369, in which IS8525 and S369 had the same structural arrangements (Suppl. Figure 1). In the second iteration, all the data were used, and the assembly results differed in contig number and connection from the first iteration. This suggests that third-generation assembly algorithms might tend to disassemble complex mitochondrial structures into more simple arrangements through overlapping relationships between long reads (Suppl. Figure 2). In the third iteration of assembly, SPAdes was used to assemble long reads which provided a more complete structural resolution given the way in which long reads are treated as k-mer fragments (Fig. 1). From these results the best final assembly strategy is to obtain stable contigs by using software for third-generation sequencing data with CLR data, and then determine structural connections between contigs by comparing these contigs with the results generated from SPAdes (Suppl. Figure 3).

**Fig. 3:**
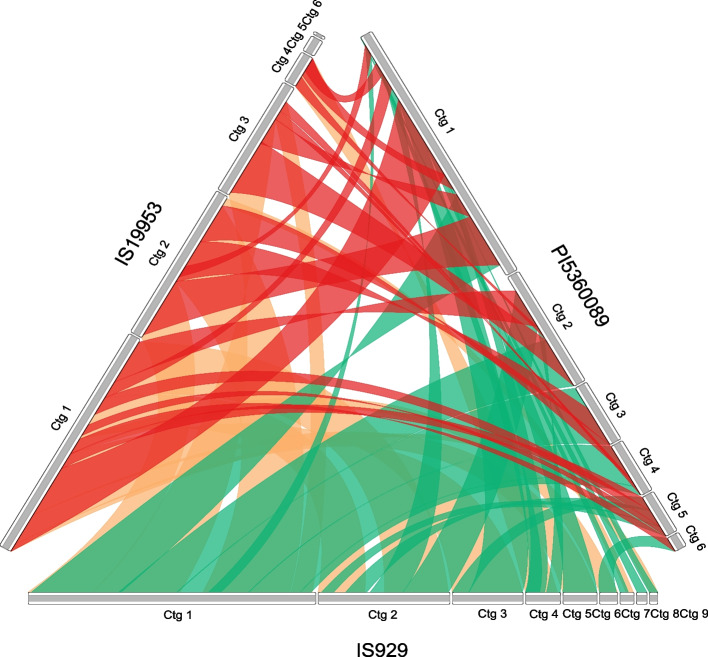
Collinearity between three sorghum mitogenomes sorted by contig. Collinear segments less than 5 kbp in length are not shown

In addition, the continuity of contigs can be used as a reference for doubling repeat sequences and confirming the connection of the two ends of the repeat sequence (Suppl. Figure 3). Each structural variant was confirmed by comparison of the three assembly methods mentioned above (Fig. 1a-c; Suppl. Figure 1–3). From this, the seven mitogenomes were divided into three types with nine or six contigs for each type. The first type includes accessions IS929, IS8525, S369 with the same structural arrangements and R931945-2–2, which differed from these by possessing two different contig linkages. The second structural type includes IS19953 and PI525695, and the third PI536008 (Fig. 1a-c). Contig connections were further verified by designing PCR primers from IS929 to confirm that the expected length from the assembly matched the PCR product length (Suppl. Table 2). The results from this test produced bands for all 12 connections, although bands from ctg6-7 and ctg9-8 were relatively faint (Fig. 1d; Suppl. Figure 19). The assembly depth of ctg7 and ctg8 was also lower than that of other contigs, suggesting that the two contigs exist in a minor conformation of the mitochondrial genome. The accessions IS929, IS19953, and PI536008 were selected to represent each structural type in collinearity analysis. All the contigs of IS929 were highly collinear with the other two accession with an alignment length greater than 5 kbp. Despite high levels of collinearity, the distributional complexity of the collinearity segments on the contigs between the three conformations shows high levels of diversity in sequence orientation within the species (Fig. 3).

Two contigs with lengths of 7,361 bp (ctg8) and 9,577 bp (ctg7) in the type I genomes and a 1,995 bp contig (ctg6) in the type II genomes were found to be without any functional genes. The depth of the two gene-free contigs in the type I genomes is lower than the surrounding contigs and may be part of secondary structural formation, while the depth of the gene-free contig in the type II genomes is much higher than the surrounding contigs and may be involved with mediation of genomic recombination (Fig. 1a-c). Furthermore, a 12.4 kbp repeat annotated in the sorghum reference mitogenome (NC_008360.1) is at a cross position in the type I and III genomes with twice the depth, while this 12.4 kbp fragment in the type II genomes is part of a longer contig (Suppl. Figure 13). Repeats annotated to the reference genome are also present in the various contigs with multi-linked relationships in the type I genomes, suggesting that such sequences are often treated as repetitive fragments in a master circle resolution of genome structure but may serve as linkages in a reticulate concept of genome structure (Suppl. Figure 14–16).

**Fig. 4:**
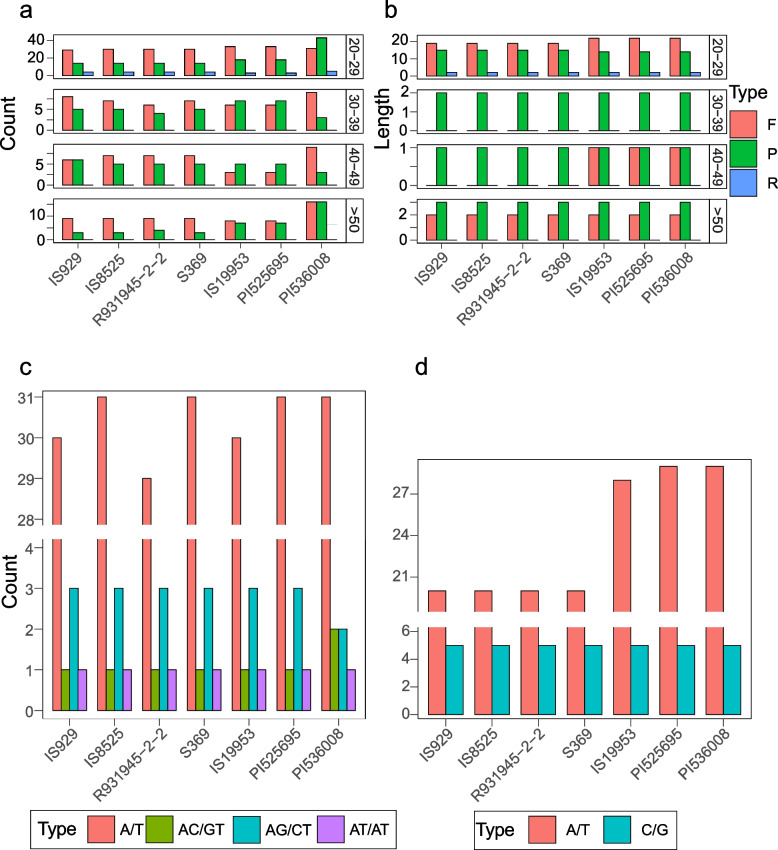
Repeat sequences of sorghum organellar genomes. **a** Mitogenome repeats identified with REPuter include F (forward repeats), R (reversed repeats), and P (palindromic repeats); **b**: Plastome repeats identified with REPuter include F, P, and R repeats; **c**: The SSRs identified from sorghum mitogenomes; **d**: The SSRs identified from sorghum plastomes

To further quantify organellar genomic structure within sorghum, repeats were annotated and compared across accessions. The mitogenomes and plastome contain three types of repeats: Forward (F), Palindromic (P), and Reverse (R). The number of repeats in the sorghum mitogenome and plastome are less frequent with increasing size (Fig. 4a, 4b; Suppl. Figure 5–12). Patterns of repeat abundance and location in the mitogenomes roughly aligned with genome structural type, where the mitogenomes from the same structural type had the same pattern of repeat sequence location in most contigs (Fig. 4a; Suppl. Figure 5–11). By contrast most of the repeat sequences in the plastomes were located in the same position in all seven plastomes (Suppl. Figure 12). Patterns of repeat abundance are less clearly associated with mitochondrial genome type in the plastome as they can be categorized into two groups containing IS929, IS8525, R931945-2–2, and S369 in one group, and the second containing IS19953, PI525695, and PI536008 (Fig. 4b; Suppl. Figure 5–12). The abundance of SSRs is relatively uniform in both plastomes and mitochondrial genomes except in the case of A/T motifs where the numbers vary more between accessions especially in plastomes (Fig. 4c and d). Moreover, the SSR diversity between plastomes and mitogenomes is markedly different with only A/T and C/G motifs present in plastomes and several different dinucleotide types present in the mitogenomes in addition to the A/T motif (Fig. 4c and d).

In the comparative analysis of the protein-coding gene sequences of mitogenomes, the CDSs of IS929, IS8525, and S369, were identically grouped by shared SNPs while IS19953, PI525695, and PI536008 were similarly grouped. The grouping of mitogenomes by CDS SNPs was exact in all cases except in R9319545-2–2 which grouped with type I genomes at all loci except for a shared SNP in *nad6* where it grouped with types II and III. Only four genes (*atp9*, *cox2*, *nad4*, and *nad6*) possess SNPs in the seven mitogenomes (Suppl. Table 3). The grouping of sorghum accessions by CDS SNPs generally matches genome structural type, with type I differentiated from types II and III wherein the largest structural differences are found. Given that R9319545-2–2 is an elite breeding line resulting from a complex parental pedigree, intermediacy in CDS SNP grouping is an expected outcome. A similar pattern separating mitogenome type I from II and III is also found among the SNPs in the plastome data yet many more SNPs were found (18 SNPs across 10 genes). The plastome data for R9319545-2–2 is similar to the mitogenome data where three of the CDS SNPs group this accession with type II and III genomes while most of the rest of the SNPs group it with the type I genomes (as well as two autapomorphic SNPs in R9319545-2–2).

### Sequence transfer from organellar genomes to the nuclear genome

**Fig. 5:**
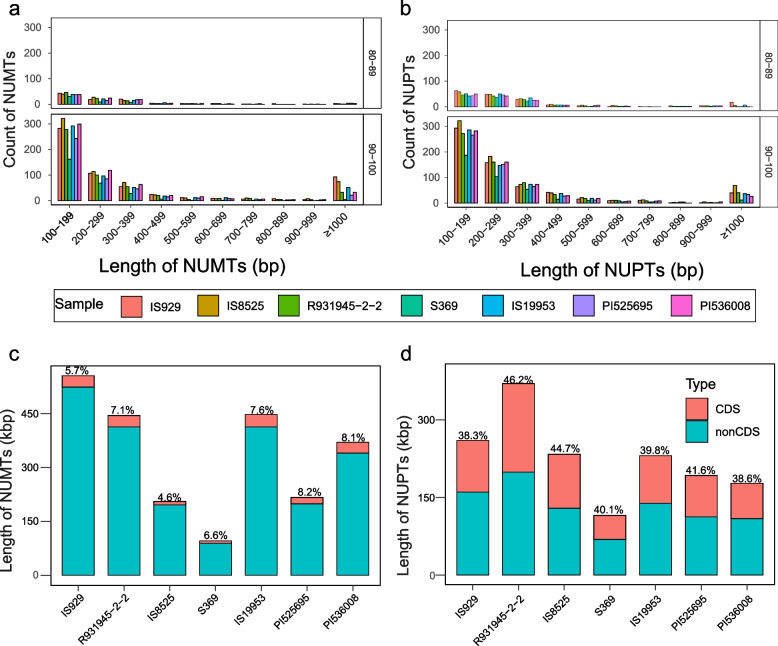
Distribution of inserts by size and type of sorghum organelle sequences transferred to the nucleus. **a** Number and length of NUMTs by accession (panels are separated by percent similarity to origin); **b**: Number and length of NUPTs by accession (panels are separated by percent similarity to origin); **c**: Total length and source sequence type of NUMTs; **d**: Total length and source sequence type of NUPTs. Percentages in the lower panels refer to CDS inserts contribution to the total length of all inserts

Intracellular sequence transfers occur frequently and continuously from organellar genomes to the nuclear genomes and are key to understanding intracellular intergenomic coevolution [16]. To investigate the patterns of intracellular sequence transfers in different sorghum accessions, the frequency and destination of sequence transfers from organellar genomes to nuclear genomes were quantified. To do this the seven sorghum organellar genomes assembled here were searched against the corresponding published nuclear genomes. The results showed that the vast majority of transferred sequences had a similarity of 90% or greater to the sequences of origin in organelles (84.4–87.5% from mitogenome, 76.1–82% from plastome) (Fig. 5a and b). The number of NUPTs is more than that of NUMTs in the 80–89% identity range, whereas they are similar in the 90–100% identity at the 100–199 bp lengths, but in all other length categories NUPTs are more abundant (Fig. 5a and b). The abundance of NUPTs and NUMTs appears to be proportional to the size of the insert with larger inserts occurring less frequently. That said several very large inserts greater than 30 kbp in length were found, with the longest NUMT and NUPT sequences being 98 kbp in PI536008 and 33 kbp in IS8525, respectively. The number of NUMTs and NUPTs in S369 was noticeably smaller than that of other accessions, which may be related to the low proportion (58%) of anchoring scaffolds to chromosomes for this nuclear genome assembly. Overall, the high abundance of transfer sequences with a sequence identity of 90–100% indicates that the organelle sequence transfer to the nucleus is a continuously ongoing process (Fig. 5a and b).

**Fig. 6:**
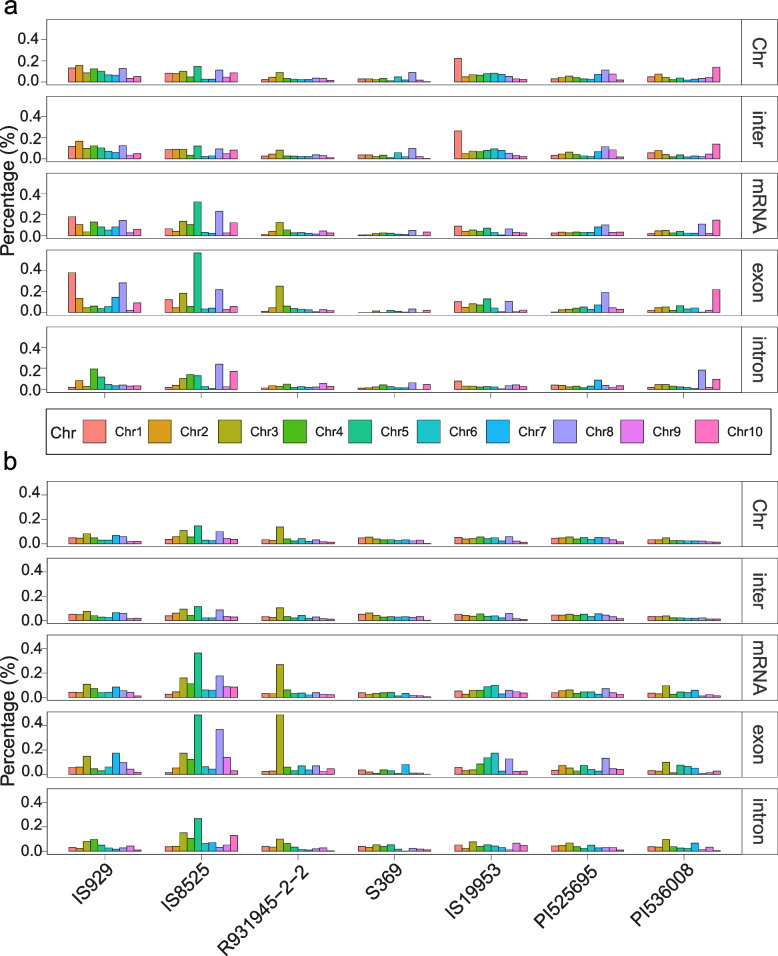
Nuclear genome locations of transferred sequences from organellar origins. **a**: The locations of NUMTs. **b**: The locations of NUPTs. Abbreviations for nuclear genome regions are Chr: Chromosome; inter: intergenic regions; mRNA: genic regions; exon: exon regions; intron: intron regions. The vertical axis represents the percentage of the length of the region into which transferred sequences reside made up of NUPTs or NUMTs

To investigate which regions from the organelles are more frequently transferred into the nuclear genome, the length of the transferred fragments from different regions (CDS and non-CDS) of the mitogenome and plastome were summed and the proportion of each type calculated for each accession. We found that the total length of NUMTs was greater than those of NUPTs except in S369 (Fig. 5c and d). However, transfers from CDSs had a higher proportion among NUPTs (4.6–8.2% for mitogenome, 38.3–46.2% for plastome) (Fig. 5c and d). In addition, the location in the nuclear genome of NUMTs and NUPTs differed by accession, chromosome, and location in the chromosome. For instance, NUMTs in most accessions consist of a roughly uniform percentage of chromosomes whereas NUMTs in IS19953 and PI536008 make up a disproportionate percentage of chromosomes 1 and 10 respectively (Fig. 6). Patterns of asymmetric NUMT insertion are also apparent when insertion location is parsed by categories such as exon and intron. In some sorghum accessions the proportion of NUMTs and NUPTs is similar, such as in the targeting of exons in chromosome 5 in IS8525 where NUMTs and NUPTs exceed 0.4%. However, in some cases NUMT and NUPT targeting appears decoupled such as in IS929, where NUMTs make up nearly 0.4% of exons in chromosome 1, whereas NUPTs account for less than 0.1% of exons in the same chromosome (Fig. 6). Overall, NUMTs and NUPTs appear to target certain locations in the nuclear genome that differ by accession.

Finally, the patterning of GC content in and around NUMTs and NUPTs was quantified to assess differences between accessions and insert types. The results indicate that the GC content of NUMTs and NUPTs is higher in sequences with 90–100% similarity to the organellar sequences of origin than those with 80–89% similarity. This is in line with expectations for the higher GC content found among plastomes and mitogenomes (Suppl. Figure 17). Similarly, the GC content among NUMTs was higher on average than NUPTs which is also expected given the higher GC content of mitogenomes over plastomes. (Suppl. Figure 17). The GC content around longer NUMTs and NUPTs was found to fluctuate more between accessions and might be related to differences in the nuclear genome related to larger inserts or might come about because of greater difficulty in determining the insert boundaries for larger inserts and thus including some of the insert as flanking sequence. (Suppl. Figure 18).

## Discussion

### Diversity in sorghum mitogenomes

Land plant mitogenomes have undergone dramatic structural changes during co-evolution in the host after endosymbiosis, while plastome structure remains strongly conserved with low levels of sequence duplication. This complex structure of plant mitogenomes poses a great challenge for accurate genome assembly [42–44]. Out of over 300 published mitogenomes, most are assigned a simplified circular structure and lack information on the structural diversity thought to occur in many mitogenomes [8]. The structures found among plant mitogenomes have been complex, and include polycyclic chromosomes, linear branches, and radial structures. Much of this diversity in structure is thought to be the result of repeat mediated recombination [45]. In addition, the structure of mitogenomes can change dynamically within a lineage or individual. For example, the structure of mitogenomes in *Vigna radiata* is linear in cold-treated seeds but rosette shaped in seeds that have not undergone cold treatment [46]. Thus, many previous studies assembling the mitogenome into a simplified master circled conformation fail to elucidate the secondary structures often present. Given this we present several different structural arrangements of the sorghum mitogenome based on iterative assembly procedures which had only previously been presented as a master circle structure in order to help guide evolutionary and functional genomics studies in the future.

In this study, we try to preserve a variety of contig linkages between stable contigs and guide the simplification of assembly results with complex network connection relationships given in DeBruijn-graph (DBG) assembly algorithm software by using reliable contigs information provided by software using a repeat graph assembly algorithm. The final mitogenome assembly presented here is resolved as a branched network structure composed of several contigs which are connected. In this study, the structure of seven accessions is divided into three types based on size and structural arrangement. The first set includes accessions IS929, IS8525, R931945-2–2, and S369, the second includes IS19953, PI525695, and the third PI536008. The inferred linkages in the mitogenome where further confirmed with PCR, using primers designed from the genome sequence of IS929 (Fig. 1a and d). In addition, PCR amplification experiments were performed in PI525695 (Type II) and PI536008 (Type III) with the 12 pairs of primers designed from IS929. The shared priming sequences in IS929 produced bands of the expected length in PI525695 (Supplementary Fig. 4a) and PI536008 (Supplementary Fig. 4b) when verified by PCR (red in Supplementary Fig. 4a), except ctg7-4 in PI525695. However, those connections unique to IS929 did not produce visible bands in PI525695 and PI536008 confirming our assembled genomic structures. Interestingly, some of the cultivated S. bicolor ssp. bicolor accessions (IS929, IS8525) share the same mitogenome structure with the wild species S. propinquum (S369), while the wild progenitor subspecies (S. bicolor ssp. verticilliflorum; PI536008) possesses a type III genome structure. The two guinea margaritiferum accessions (IS19953, PI525695), which represent the second domestication event from West Arica, both possess a type II genome structure. It is possible that mitogenome structure could be associated with certain domesticated traits, but further work is needed to confirm this possible relationship (Fig. 1a-c). In addition, the contigs of the final conformation in IS19953 can be well connected in the de novo assembled network conformation of the PI536008 when using the NGS data, indicating that the mitogenome of PI536008 might be the same as IS19953 in the second conformation. However, differences in size and structure of mitochondrial sequences in the assembled mitogenomes presented here may in part be due to the limitations of existing software, which need to be further refined for specifically resolving the complex arrangements of plant mitogenomes [47]. Overall, the classification results of sorghum mitogenomes and plastomes were consistent.

### Organellar sequence transfer to the nucleus

Extant plant mitochondria and plastids contain far fewer genes than their ancestors, reflecting the migration of genes from organelles to the nucleus [48–52]. The transfer of genetic material from organelles to the nucleus has been unceasing through time and has mainly involved the transfer of non-functional DNA [53]. Although many recent organelle-derived sequences are inactive or nonfunctional, exceptions exist, and some of these functional transfers have had implications for cellular metabolism and genomic evolution [17]. In the analysis of organelle transfers into the nucleus, we observed a 90–100% identity for most of the transferred sequences, indicating that the transfer of these sequences has been sustained and has occurred up to the present. Moreover, these newly transferred sequences still maintain a GC content similar to that of the source organellar genome, indicating that these fragments retain the structure of the original sequence, while the transferred sequences with 80–89% identity have a lower GC content than both the source genome and the nuclear genome, suggesting that these transferred fragments may undergo alternative fates than the surrounding nuclear genomic DNA, including differing rates of elimination, mutation, rearrangement, fragmentation, or proliferation (Suppl. Figure 17–18). Previous studies regarding plastid and mitochondrial DNA insertions in the nucleus (NUPTs and NUMTs) found patterns of asymmetric deposition [54]. Here too, we found differing rates of deposition for NUMTs and NUPTs as a function of percent of the region in which the transfers reside and the accession that was examined which could be the result of both assembly quality and biological processes. Therefore, future studies should explore how organellar transfers in sorghum could be involved with tolerance to environmental stress and potentially how this process could be harnessed to improve cultivars to withstand adverse growing conditions.

### Coevolution of mitogenome structure and sequence in sorghum

Plant mitochondrial DNA has been described as evolving rapidly in structure, but very slowly in coding sequence [55]. Numerous studies on the evolution of the coding sequences have confirmed the extremely low rate of nucleotide substitutions in plant mitogenome CDSs relative to other eukaryotic organelles [56–58]. When comparing the CDSs of sorghum mitogenomes, we found that only four of 32 genes (*nad4*, *nad6*, *atp9*, and *cox2*) had a single SNP difference between accessions, which splits the accessions into two groups based on shared SNPs. However, R931945-2–2 is intermediate between the groups as it clusters with type I genomes on all but one SNP (*nad6*) where it clusters with type II and III genomes. This outlier status for R931945-2–2 is further confirmed in the plastome CDS data where it often shares SNPs with type I genomes but occasionally shares SNPs with type II and III genomes as well as possessing unique SNPs. Structurally R931945-2–2 differs from other type I genomes in the fusion of two contigs (ctg4 and ctg5) that were resolved separately in other type I genomes and the presence of an additional contig (ctg8, 8900 bp) (Fig. 1; Suppl. Table 3-4). In addition, repeat sequence abundance provides a means to separate the different genome types especially the longer repeats in the mitogenome (Fig. 4). The R931945-2–2 accession is an elite breeding line developed from a pre-breeding program in Australia and is referred to as a ‘complex’ cultivar in Tao et al. 2021 reflecting its complex pedigree [59]. As such the unique mitogenome structure and intermediacy of SNPs in this accession may have come about from extensive heteroplasmy associated with breeding between diverse lineages [60, 61]. In addition, the grouping of structural types and SNP diversity among mitogenomes appears to match the two origins for sorghum in east and west Africa [59]. The type I genomes contain cultivars from East Africa and a closely related wild species (*S. propinquum*, S369) suggesting that there may be a relationship between cultivars and wild relatives, such as hybridization and introgression, while the type II genomes represent the second domestication event in West Africa.

## Conclusion

Before this publication only one simplified master circle reference mitogenome assembled using NGS data and four plastomes were publicly available for sorghum. Here, we assembled the reticular mitogenomes of *S. propinquum* (S369) and six accessions of *S. bicolor* (IS929, IS8525, R931945-2–2, IS19953, PI525695, PI536008) from different cultivars and wild sources with long PacBio CLR reads and NGS data. The resolution of reticular mitogenomes improves our understanding of organellar genome structure in sorghum and provides a template for further research into questions such as how gene function could be mediated by different structural conformations. In the use of long PacBio CLR reads and NGS data for mitogenome assembly, we propose that by using the reliable contigs information provided by a repeat graph assembly algorithm to guide the assembly results with complex contigs provided by a DBG algorithm will be helpful in resolving complex genomic structures in other plant species. We found inter-sequence differences between sorghum accessions discovered using the collinearity of mitogenome sequences, as well as SNPs in the CDSs in both mitogenomes and plastomes. Such data will be useful in screening phenotypes associated with a given genomic and/or nucleotide feature for later use in breeding and cultivar improvement. Analysis of NUMTs and NUPTs in the nuclear genome showed that most of the transferred sequences were recent and short in length with differences in nuclear genome deposition by genomic region and accession. This pattern of differential deposition into the nuclear genome should be followed-up on to gain a better understanding of how such transfers were involved with the domestication process and could be utilized in the future for cultivar improvement.

## Methods

### Samples

A total of seven sorghum accessions (Table 1) were selected from Tao et al. and its corresponding Illumina and PacBio CLR reads were downloaded from the project CNP0001440 in the China National GeneBank database (https://db.cngb.org) [32]. The assembly and annotation information of the seven sorghum genomes were obtained from SORGHUMBASE [32].

### Genome assembly and annotation

Organelle genome assembly was completed using the method of mitogenome assembly from Hong et al. [13]. In the first step, ~ 8 Gbp of short reads were randomly extracted from the Illumina data of seven sorghum genomes, and SPAdes v3.15.2 [62] was used to generate a draft mitochondrial genome, with the parameter settings ‘-careful -k 21,51,71,91,101 -cov-cutoff auto’. Blast v2.2.21 [63] in Bandage v 0.8.1 [64] was used to align the published sorghum mitogenome (NCBI accession number: NC_008360.1) and the reference plastome (NCBI accession number: NC_008602.1) onto seven mitochondrial and plastid drafts respectively. The 3.2Mbp of contigs for the mitogenomes were further extracted as ‘baits’. Subsequently, in the second step approximately 10 Gbp of PacBio CLR reads were randomly extracted for each of the seven sorghum genomes. The mitochondrial contigs obtained in the first step were used as baits to extract a total 118 of Mbp–285 Mbp mitochondrial CLR reads for each sorghum accession by Blastn v2.11.0 + [63] with the parameter settings ‘-evalue 1e-6’. These were further corrected by the correct subroutine in NextDenovo v2.4.0 (https://github.com/Nextomics/NextDenovo), and finally 26 Mbp–43 Mbp of self-corrected CLR reads were obtained. Flye-meta v2.8.3-b1695 [65] was used to assemble the seven mitogenomes with self-corrected CLR reads, and two to nine mitochondrial contigs in the range of 386 kbp to 453 kbp were obtained. In order to obtain more mitochondrial data, the pipeline used in the second step was repeated, but in the third step all CLR reads from seven sorghum accessions were used to select CLR mitochondrial reads, with 118 Mbp–445 Mbp of reads obtained. Then the correct subroutine in NextDenovo v2.4.0 (https://github.com/Nextomics/NextDenovo) was also used to obtain 26–53 Mbp self-corrected CLR reads. Flye-meta v2.8.3-b1695 [65] was used to assemble the seven mitogenomes with self-corrected CLR reads, and two to nine mitochondrial contigs in the range of 394 kbp to 440 kbp were obtained. In step four, SPAdes v3.15.2 [62] was used for de novo assembly of seven mitogenomes by self-corrected CLR reads that were obtained in step three, with the parameter settings ‘-careful -k 21,51,71,91,101 -cov-cutoff auto -phred-offset 33’. Bandage v0.8.1 [64] was used to remove non-mitochondrial sequences in the reticular assembly results of the obtained mitogenomes, and then the embedded Blastn v2.11.0 + [63] was used to compare the mitochondrial contigs obtained in step two to the clean reticular assembly with a total of six or nine contigs per mitogenome with sizes from 396–445 kbp. Pilon v1.23 [66] was used for three rounds of polishing with Illumina reads from which a final mitochondrial assembly was obtained.

For the chloroplast genome assembly, the chloroplast graph was extracted and then manually assembled into the final circular structure from the de novo assembly results of the Illumina reads mentioned above with Bandage v0.8.1 [64], based on the reference chloroplast genome of *S. bicolor* (NCBI accession number: NC_008602.1).

The online tool GeSeq (https://chlorobox.mpimp-golm.mpg.de/geseq.html) [67] was used to annotate the contents for each contig of each mitochondrial genome, based on the reference mitochondrial genome of *S. bicolor* (NCBI accession number: NC_008360.1). For the parts of genes annotated into different contigs, we spliced and annotated them manually. The chloroplast genome was also annotated by the GeSeq (https://chlorobox.mpimp-golm.mpg.de/geseq.html) [67] and PGA [68] software, by using the reference chloroplast genome of *S. bicolor* (NCBI accession number: NC_008602.1).

### PCR amplification to confirm mitochondria genome structure

Bandage v0.8.1 [64] was used to merge contigs with pairwise connections and generate a single connected sequence, based on the mitogenome conformation resolved for IS929. Then Primer Premier 6 (Premier Biosoft Interpairs, Palo Alto, CA) was used to design primers in the range of 0.5–2 kbp on both sides of each linkage site for each linkage variant. The DNA isolated from young leaf tissue of *S. bicolor* ssp. *bicolor* (IS929) was used to conduct PCR verification. PCR amplification products that crossed linkage sites were then used to verify each linkage relationship (Suppl. Table 2). PCRs were performed in volumes of 20 uL consisting of 1 uL template DNA, 0.4 uL 2.5 mM dNTP, 2 uL 10 × EasyTaq® Buffer (TransGen), 0.2 uL 500U EasyTaq® DNA Polymerase (TransGen), 0.2uL 100uM forward primer, 0.2uL 100uM reverse primer, and 16 uL ddH_2_O. Thermocycling conditions were 95℃ denaturation for three minutes, followed by 35 cycles each including 95℃ denaturation for 30 s, 60 ~ 61.5 ℃ annealing for 30 s (60 ℃ for ctg9-8; 60.5 ℃ for ctg1-5, 2–4, 3–5, 4–1, 6–3, 6–7, 7–4, 8–6, 9–4, 9–5; 61.5 ℃ for ctg6-2), and 72℃ extension for three minutes. Following the 35 cycles a final 5-min extension step at 72℃ was conducted. The PCR products were assessed for length using a 1.5% agarose gel run at 100 V for 25 min and compared to an 8 kbp ladder.

### Sequence alignment of protein-coding genes

The CDS of the 32 mitochondrial and 76 chloroplast PCGs for each accession were extracted and aligned for each gene using MAFFT v7.490 for SNP identification [69, 70]. These alignments were used to detect and group differences among the different sorghum accessions.

### Analysis of intracellular transfers

Blastn v2.11.0 + [63] was used to identify transfer events from organelles to the nuclear genome with the filter parameter ‘identity greater than 80% and alignment length greater than 100 bp’. These results were further divided into two datasets: 80–89% and 90–100% similarity to a known organelle sequence to represent older (more mutations) and newer (fewer mutations) transfers respectively. In addition, each dataset was further divided into different length categories of 100–199 bp, 200–299 bp, 300–399 bp, 400–499 bp, 500–599 bp, 600–699 bp, 700–799 bp, 800–899 bp, 900–999 bp, and 1000 bp and above. BEDtools v2.30.0 [71] was used to annotate organelle transfer locations in the nuclear genome and their distribution patterns in different chromosomes and genomic regions (i.e. exon, intron, intergenic, mRNA) based on the annotation file of the nuclear genome. The transfer fragment and the 5’ and 3’ flanking sequences (100–499 bp, 500–1000 bp, and above 1000 bp) were extracted and calculated for GC content. The program ggplot2 v3.3.6 [72] was used for visualization.

### Repeat sequence detection

Four dispersed repeat sequence types were assessed for both organelles of seven sorghum accessions. The repeat types, F (forward), P (palindrome), R (reverse), and C (complement) were detected using REPuter [73] with default parameters. The location of the three type repeats in the organelle genomes were plotted using the online website tool MG2C_v2.1 [74]. The MISA software [75] was used to identify simple sequence repeats (SSRs) with 10, 6, 5, 5, 5, and 5 repeat units set as minimum thresholds for mono-, di-, tri-, tetra-, penta-, and hexa-motifs respectively.

## Supplementary Information

**Additional file 1: Supplementary Table 1.** Genome contents of plastomes assembled in this study. **Supplementary Table 2.** The primer designs to confirm the 12 connections of the Type I conformation in IS929. **Supplementary Table 3.** SNPs in the CDSs of sorghum mitogenomes. **Supplementary Table 4.** SNPs in the CDSs of sorghum plastomes.

**Additional file 2: Supplementary Fig. 1.** Mitogenome structure of seven sorghum accessions whenusing 10G of CLR data. **Supplementary Fig. 2.** Mitogenome structure of seven sorghum accessions when using all available CLR data. **Supplementary Fig. 3.** Example of the final mitogenome conformation process. The conformation is from the mitogenome assembled by SPAdes using corrected CLR reads of IS929, the color-coded portion is contig-01 of IS929 assembled by Flye using 10G CLR, which were compared to the conformation, from red to pink, representing the continuity of the contig. **Supplementary Fig. 4.** PCR amplification to check the 12 linkages of IS929(Type I) in PI525695(Type II) and PI536008(Type III). The colors and labels in a and b show the same connections of contigs of IS929 in PI525695 and PI536008. The red marked labels in c are corresponding to the connections in **a** and **b**. **Supplementary Fig. 5.** The location of forward repeats(F), reversed repeats(R) and P palindromic repeats(P) in the contig-01 of seven sorghum mitogenomes. **Supplementary Fig. 6.** The location of forward repeats (F), reversed repeats (R) and P palindromic repeats (P) in the contig-02 of seven sorghum mitogenomes. **Supplementary Fig. 7.** The location of forward repeats (F), reversed repeats (R) and P palindromic repeats (P) in the contig-03 of seven sorghum mitogenomes. **Supplementary Fig. 8.** The location of forwardrepeats (F), reversed repeats (R) and P palindromic repeats (P) in the contig-04 of seven sorghum mitogenomes. **Supplementary Fig. 9.** The location of forward repeats (F), reversed repeats (R) and P palindromic repeats (P) in the contig-05 of seven sorghum mitogenomes. **Supplementary Fig. 10.** The location of forward repeats (F), reversed repeats (R) and P palindromic repeats (P) in the contig-06 of seven sorghum mitogenomes. **Supplementary Fig. 12.** The location of forward repeats (F), reversed repeats(R) and P palindromic repeats (P) in seven sorghum plastid genomes. **Supplementary Fig. 13.** Location of a 12.4 kbp repeat sequence from sorghum reference genome NC_008360.1 in each of three structural types. **Supplementary Fig. 14.** Location of a 3.6 kbp repeat sequence from sorghum reference genome NC_008360.1 in each of three structural types. **Supplementary Fig. 15.** Location of a 4 kbp repeat sequence from sorghum reference genome NC_008360.1 in each of three structural types. **Supplementary Fig. 16.** Location of a 33 kbp repeat sequence from sorghum reference genome NC_008360.1 in each of three structural types. **Supplementary Fig. 17.** GC content of fragments transferred from sorghum organelles to the nucleus. **a**: GC content of NUMTs with different levels of sequence identity; **b**: GC content of NUPTs with different levels of sequence identity. **Supplementary Fig. 18.** The GC content of transfer fragment flanking sequences **a**: The GC content of NUMT flanking sequences. **b**: The GC content of NUPT flanking sequences. **Supplementary Fig. 19.** The full-length original gel of Figure 1d. 

## Data Availability

The mitogenome and plastome sequences supporting the conclusions of this article are available in GenBank (https://www.ncbi.nlm.nih.gov/) with accession numbers: OP474086-OP474139 for mitogenome, OP474079-OP474085 for plastome.

## Abbreviations

Mitogenomes: Mitochondrial genomes
plastomes: Plastid genomes
ETC: Electron transport chain
NUMTs: Nuclear mitochondrial DNAs
NUPTs: Nuclear plastid DNAs
CMS: Cytoplasmic male sterility
LSC: Large single copy region
SSC: Small single copy region
IR: Inverted repeats
PCGs: Protein-coding genes
SSRs: Simple sequence repeats
CDSs: Coding sequences
DBG: DeBruijn-graph
NCBI: National Center for Biotechnology Information
