# Supplementary material for: Variation in mitogenome structural conformation in wild and cultivated lineages of sorghum corresponds with domestication history and plastome evolution
Source: BMC Plant Biol. 2023 Feb 13;23:91. doi: 10.1186/s12870-023-04104-2 (PMC9926791; doi:10.1186/s12870-023-04104-2)
Supplement: Supplementary file 2 — Additional file 2: Supplementary Fig. 1. Mitogenome structure of seven sorghum accessions whenusing 10G of CLR data. Supplementary Fig. 2. Mitogenome structure of seven sorghum accessions when using all available CLR data. Supplementary Fig. 3. Example of the final mitogenome conformation process. The conformation is from the mitogenome assembled by SPAdes using corrected CLR reads of IS929, the color-coded portion is contig-01 of IS929 assembled by Flye using 10G CLR, which were compared to the conformation, from red to pink, representing the continuity of the contig. Supplementary Fig. 4. PCR amplification to check the 12 linkages of IS929(Type I) in PI525695(Type II) and PI536008(Type III). The colors and labels in a and b show the same connections of contigs of IS929 in PI525695 and PI536008. The red marked labels in c are corresponding to the connections in a and b. Supplementary Fig. 5. The location of forward repeats(F), reversed repeats(R) and P palindromic repeats(P) in the contig-01 of seven sorghum mitogenomes. Supplementary Fig. 6. The location of forward repeats (F), reversed repeats (R) and P palindromic repeats (P) in the contig-02 of seven sorghum mitogenomes. Supplementary Fig. 7. The location of forward repeats (F), reversed repeats (R) and P palindromic repeats (P) in the contig-03 of seven sorghum mitogenomes. Supplementary Fig. 8. The location of forwardrepeats (F), reversed repeats (R) and P palindromic repeats (P) in the contig-04 of seven sorghum mitogenomes. Supplementary Fig. 9. The location of forward repeats (F), reversed repeats (R) and P palindromic repeats (P) in the contig-05 of seven sorghum mitogenomes. Supplementary Fig. 10. The location of forward repeats (F), reversed repeats (R) and P palindromic repeats (P) in the contig-06 of seven sorghum mitogenomes. Supplementary Fig. 12. The location of forward repeats (F), reversed repeats(R) and P palindromic repeats (P) in seven sorghum plastid genomes. Supplementary Fig. 13. Location o [file 12870_2023_4104_MOESM2_ESM.docx]

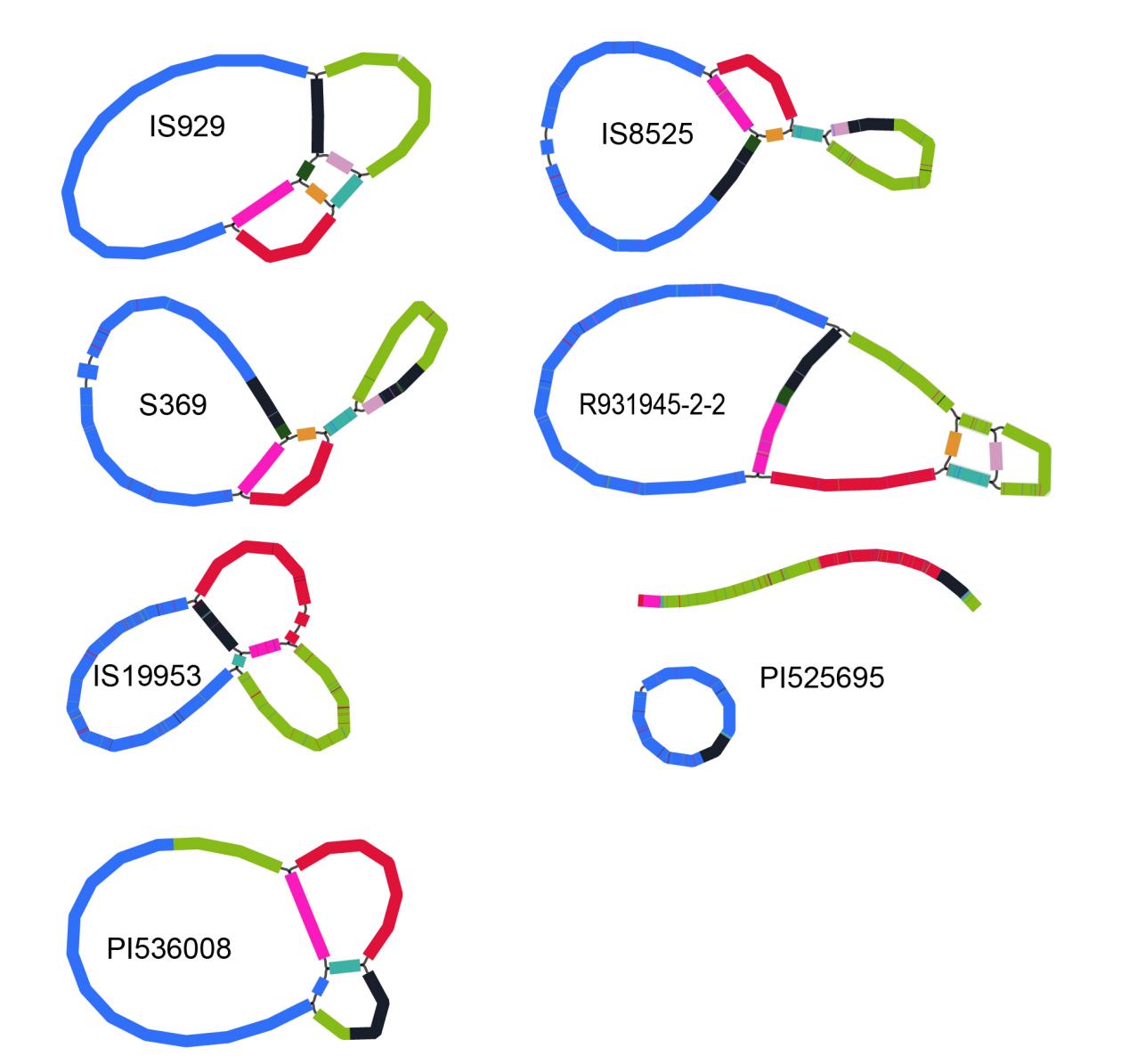


**Supplementary Fig. 1.** Mitogenome structure of seven sorghum accessions when using 10G of CLR data.
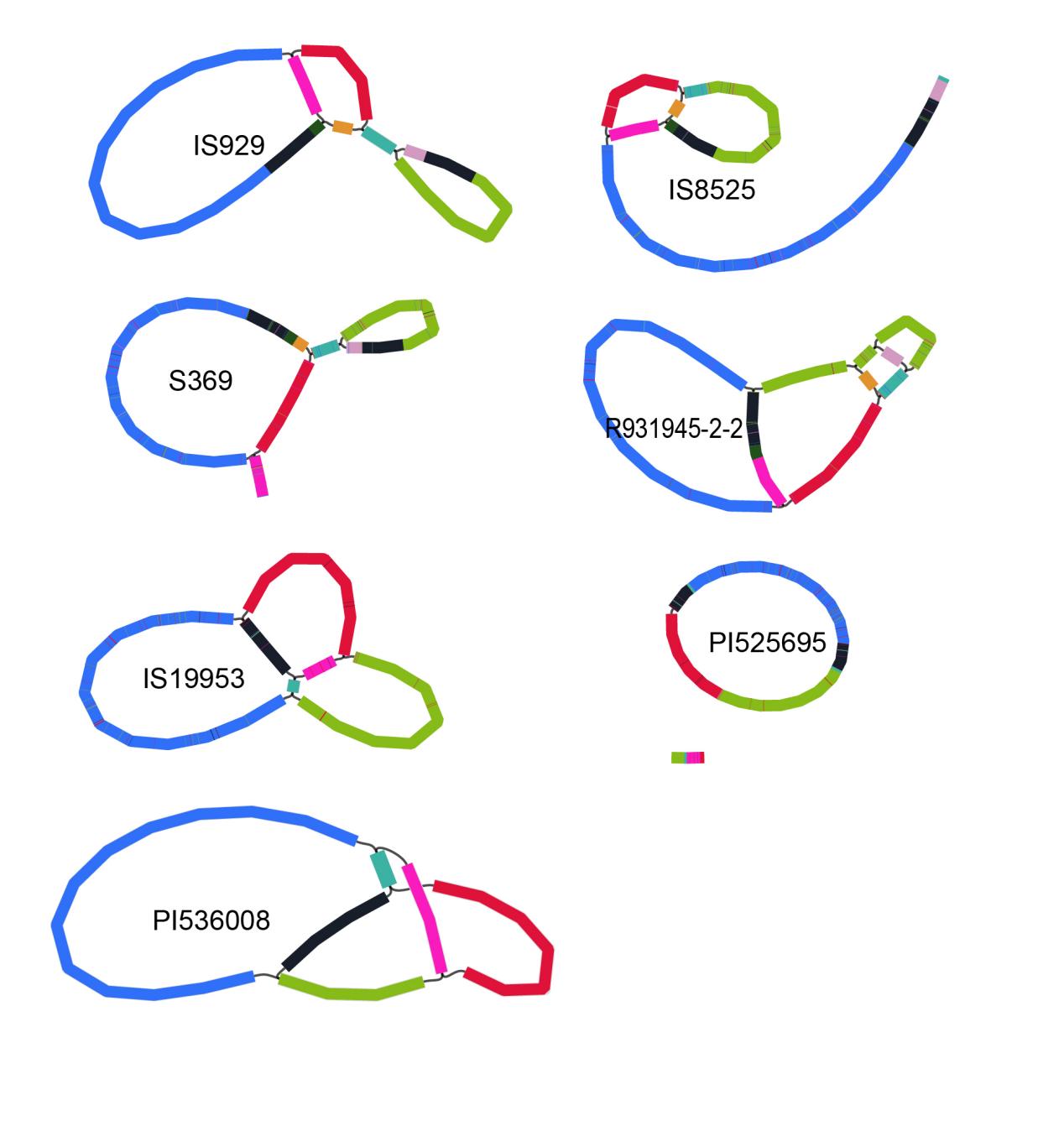
**Supplementary Fig. 2.** Mitogenome structure of seven sorghum accessions when using all available CLR data.


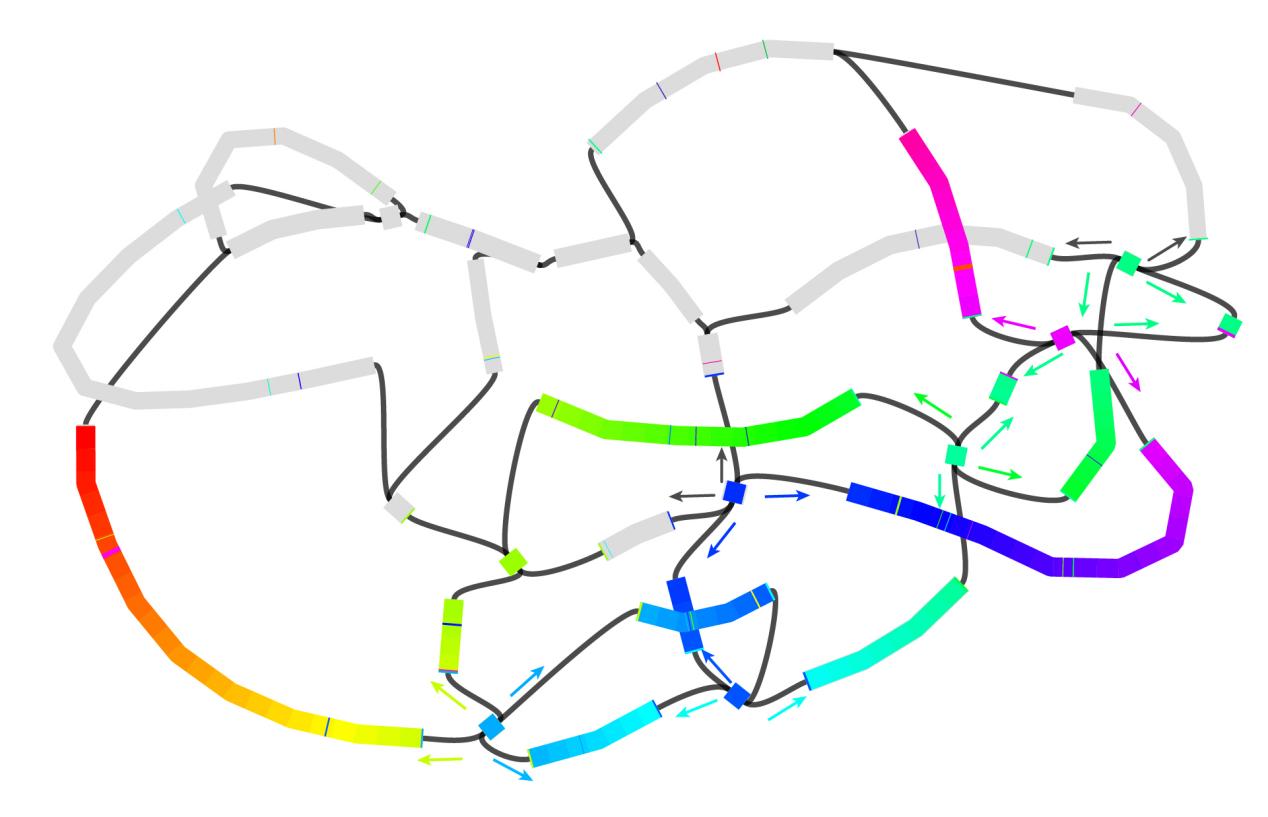


**Supplementary Fig. 3.** Example of the final mitogenome conformation process. The conformation is from the mitogenome assembled by SPAdes using corrected CLR reads of IS929, the color-coded portion is contig-01 of IS929 assembled by Flye using 10G CLR, which were compared to the conformation, from red to pink, representing the continuity of the contig.

**Supplementary Fig. 4.** PCR amplification to check the 12 linkages of IS929(Type I) in PI525695(Type II) and PI536008(Type III). The colors and labels in a and b show the same connections of contigs of IS929 in PI525695 and PI536008. The red marked labels in c are corresponding to the connections in a and b.
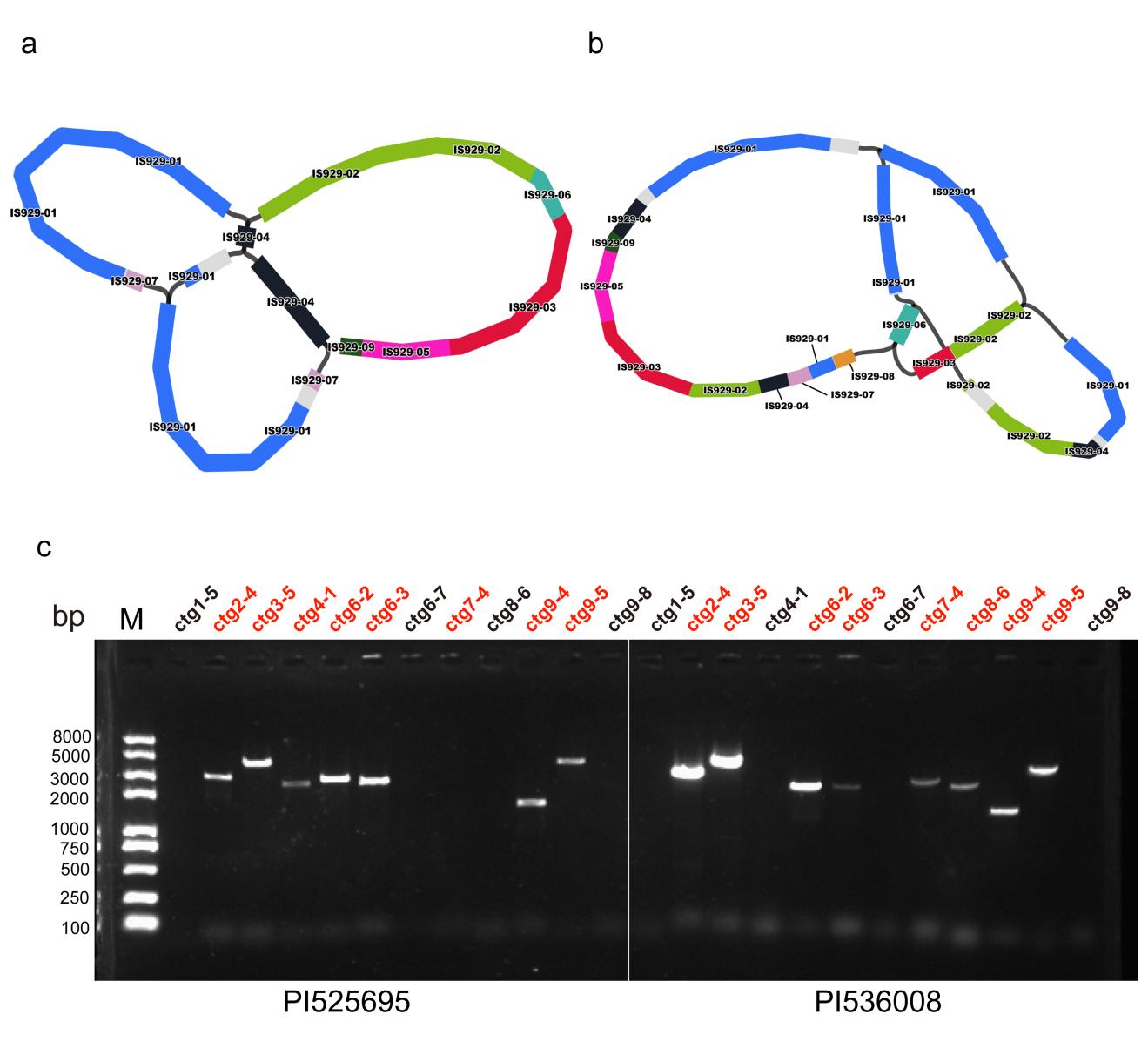


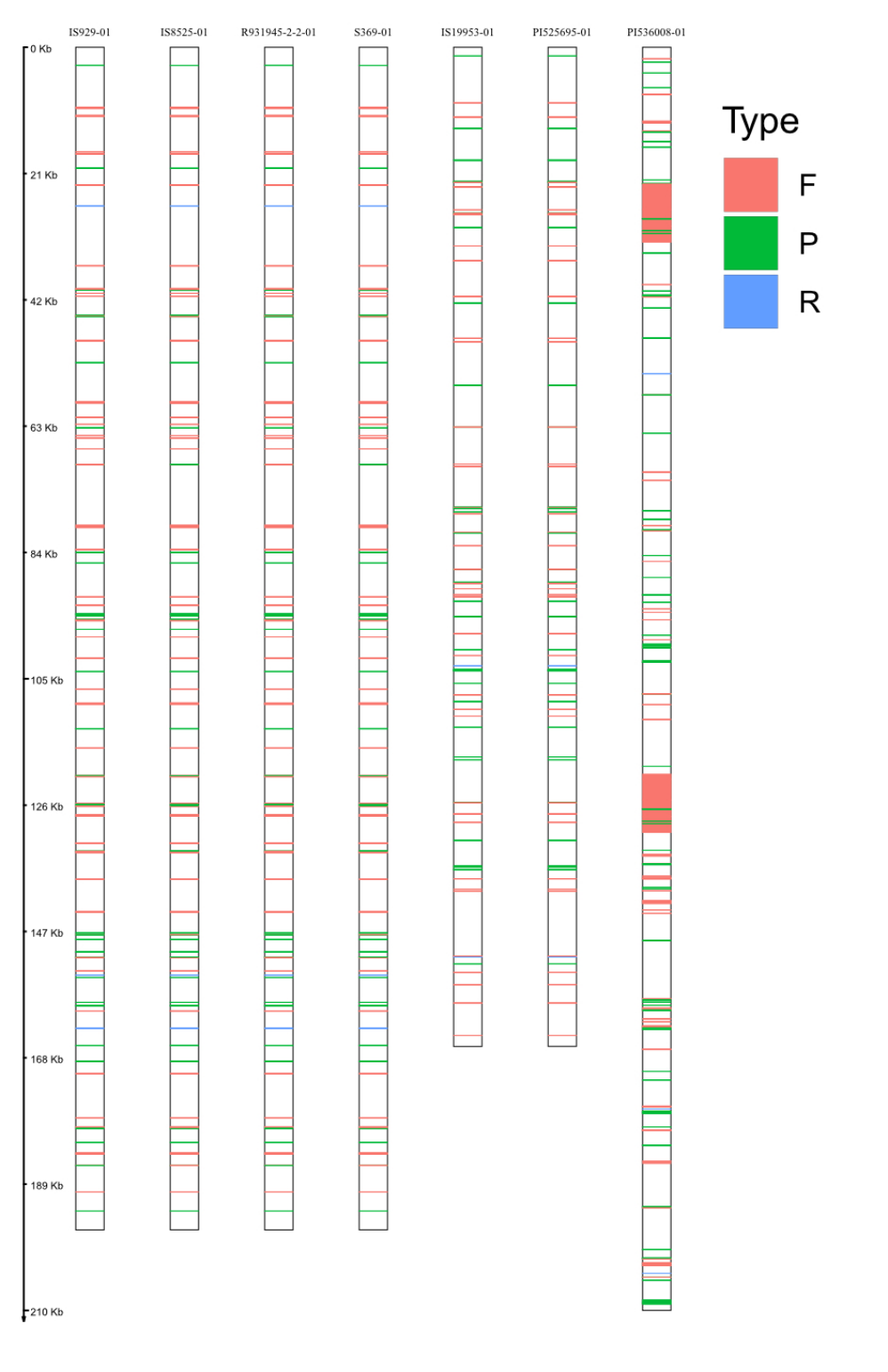
**Supplementary Fig. 5.** The location of forward repeats(F), reversed repeats(R) and P palindromic repeats(P) in the contig-01 of seven sorghum mitogenomes.

**Supplementary Fig. 6.** The location of forward repeats (F), reversed repeats (R) and P palindromic repeats (P) in the contig-02 of seven sorghum mitogenomes.
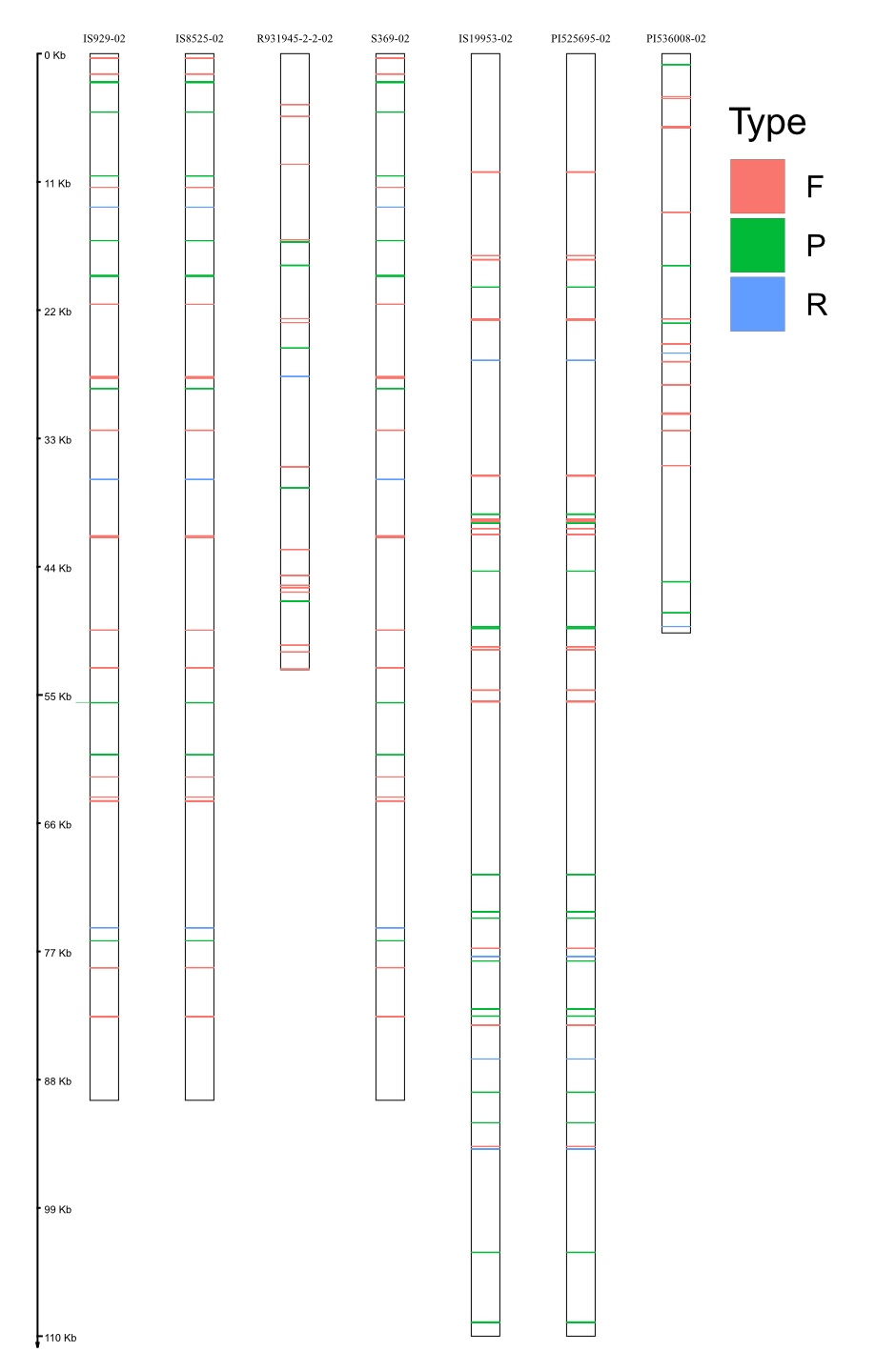


**Supplementary Fig. 7.** The location of forward repeats (F), reversed repeats (R) and P palindromic repeats (P) in the contig-03 of seven sorghum mitogenomes
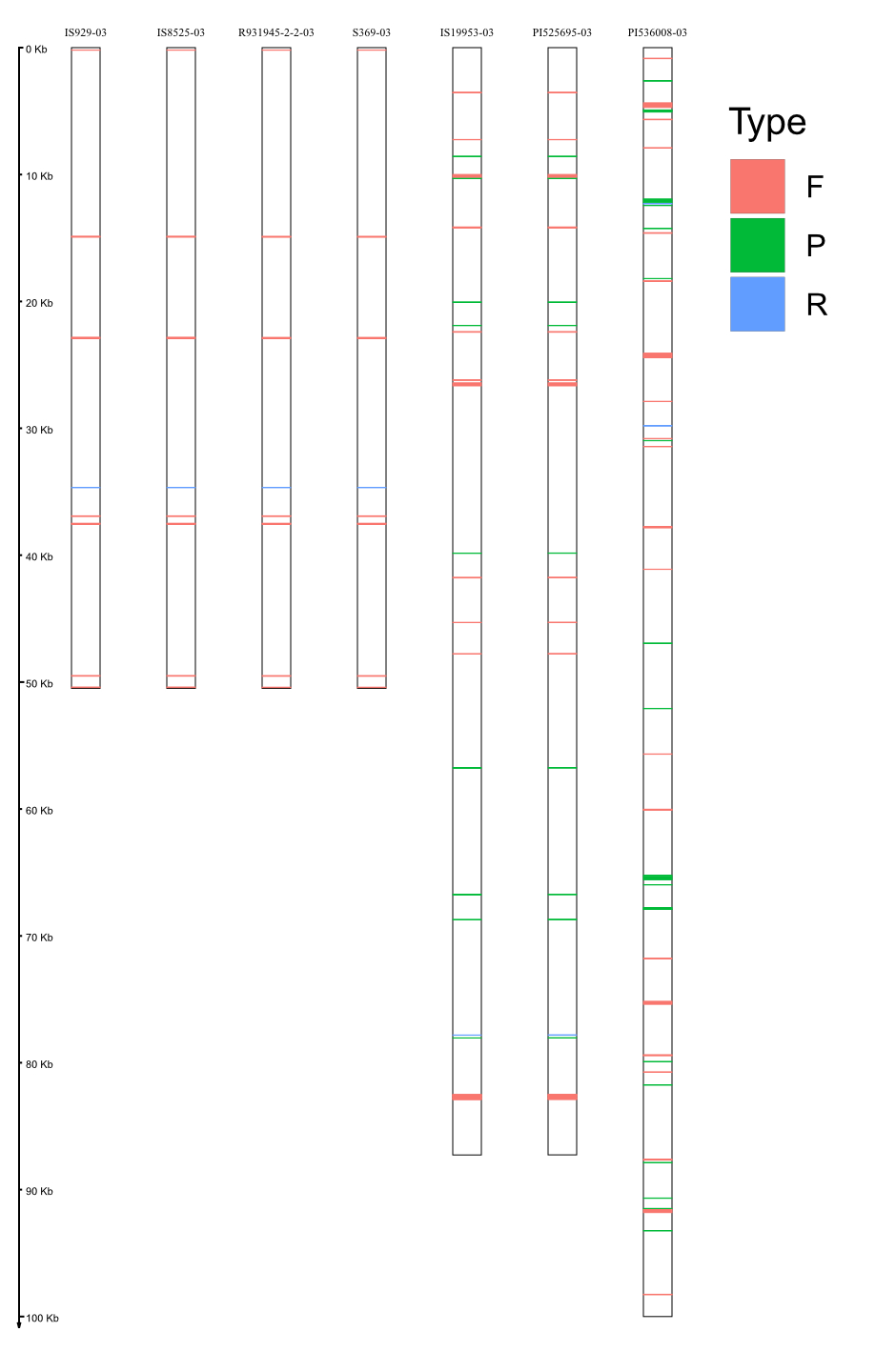
.

**Supplementary Fig. 8.** The location of forward repeats (F), reversed repeats (R) and P palindromic repeats (P) in the contig-04 of seven sorghum mitogenomes.
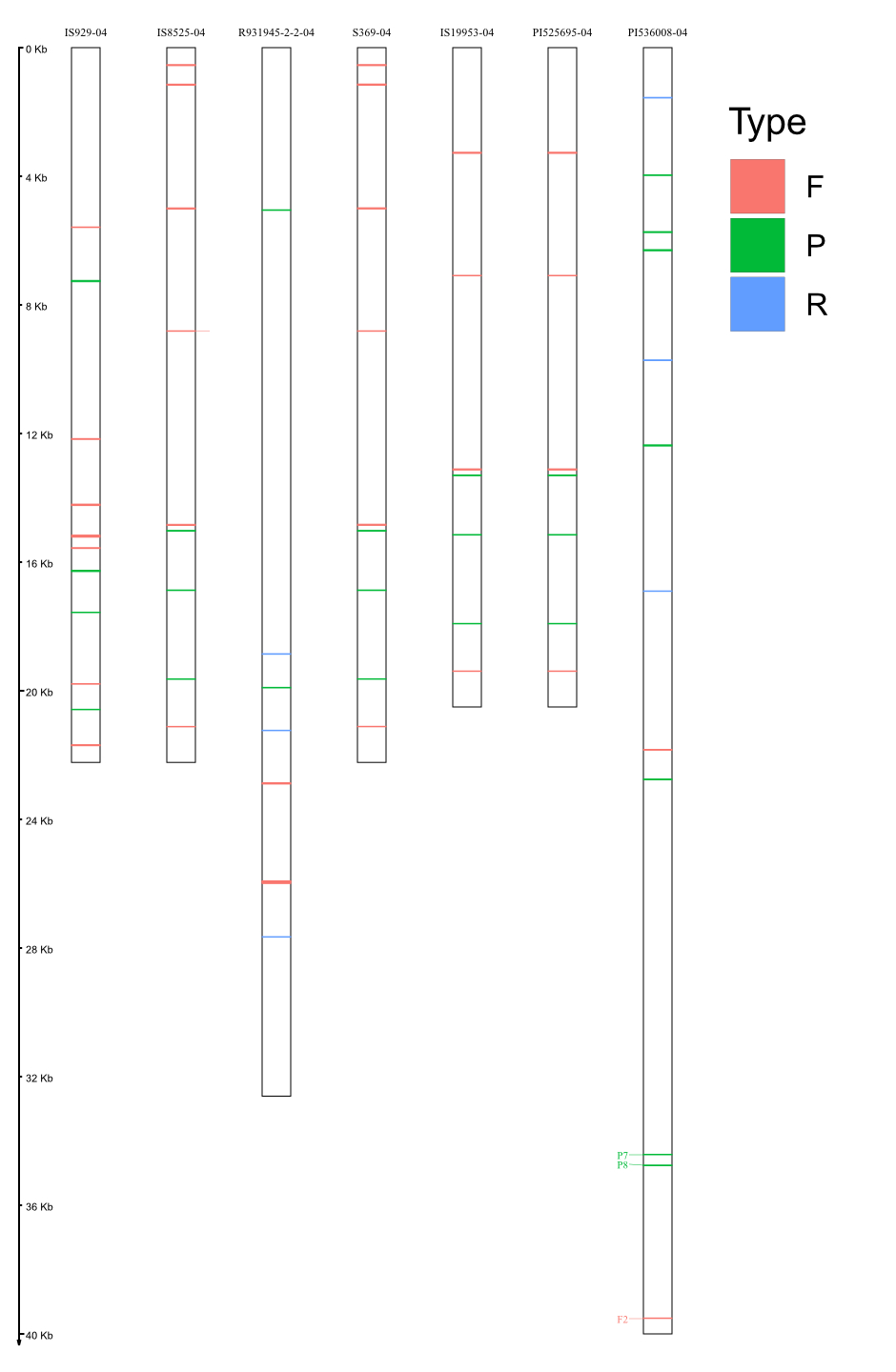


**Supplementary Fig. 9.** The location of forward repeats (F), reversed repeats (R) and P palindromic repeats (P) in the contig-05 of seven sorghum mitogenomes.
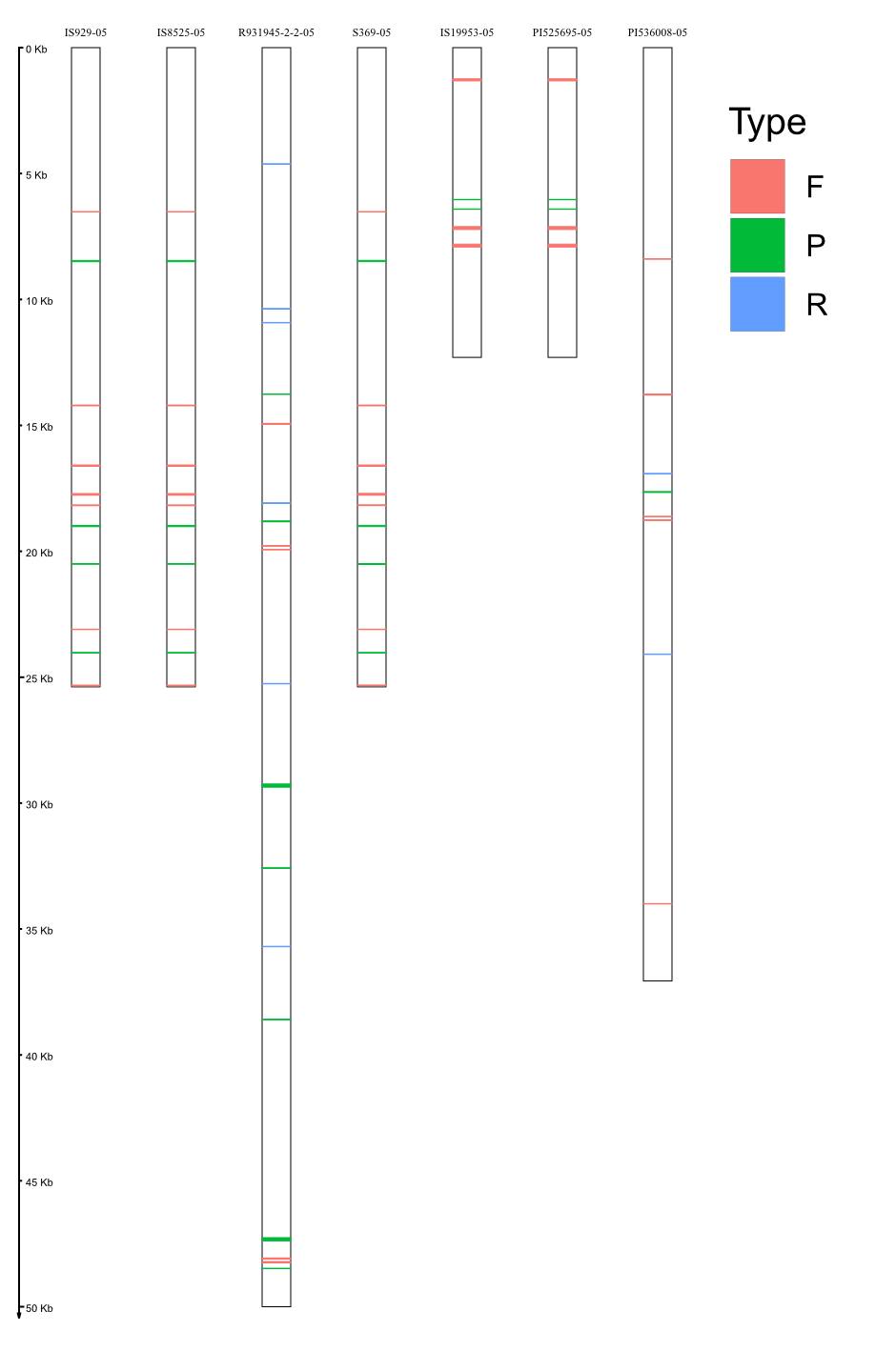


**Supplementary Fig. 10.** The location of forward repeats (F), reversed repeats (R) and P palindromic repeats (P) in the contig-06 of seven sorghum mitogenomes.
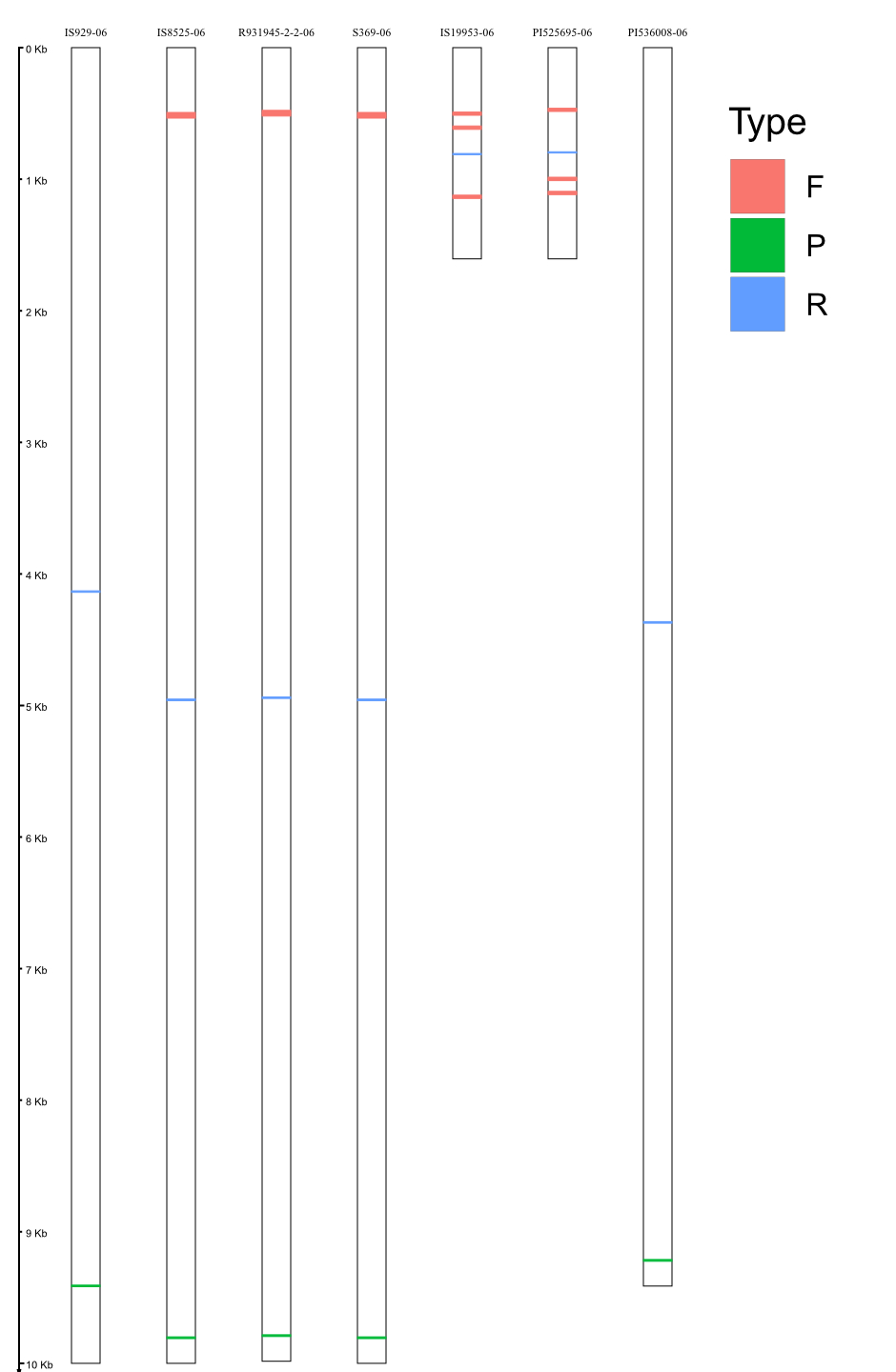


**Supplementary Fig. 11.** The location of forward repeats (F), reversed repeats (R) and P palindromic repeats (P) in the contig-07,- 08, -09 of type I sorghum mitogenomes.
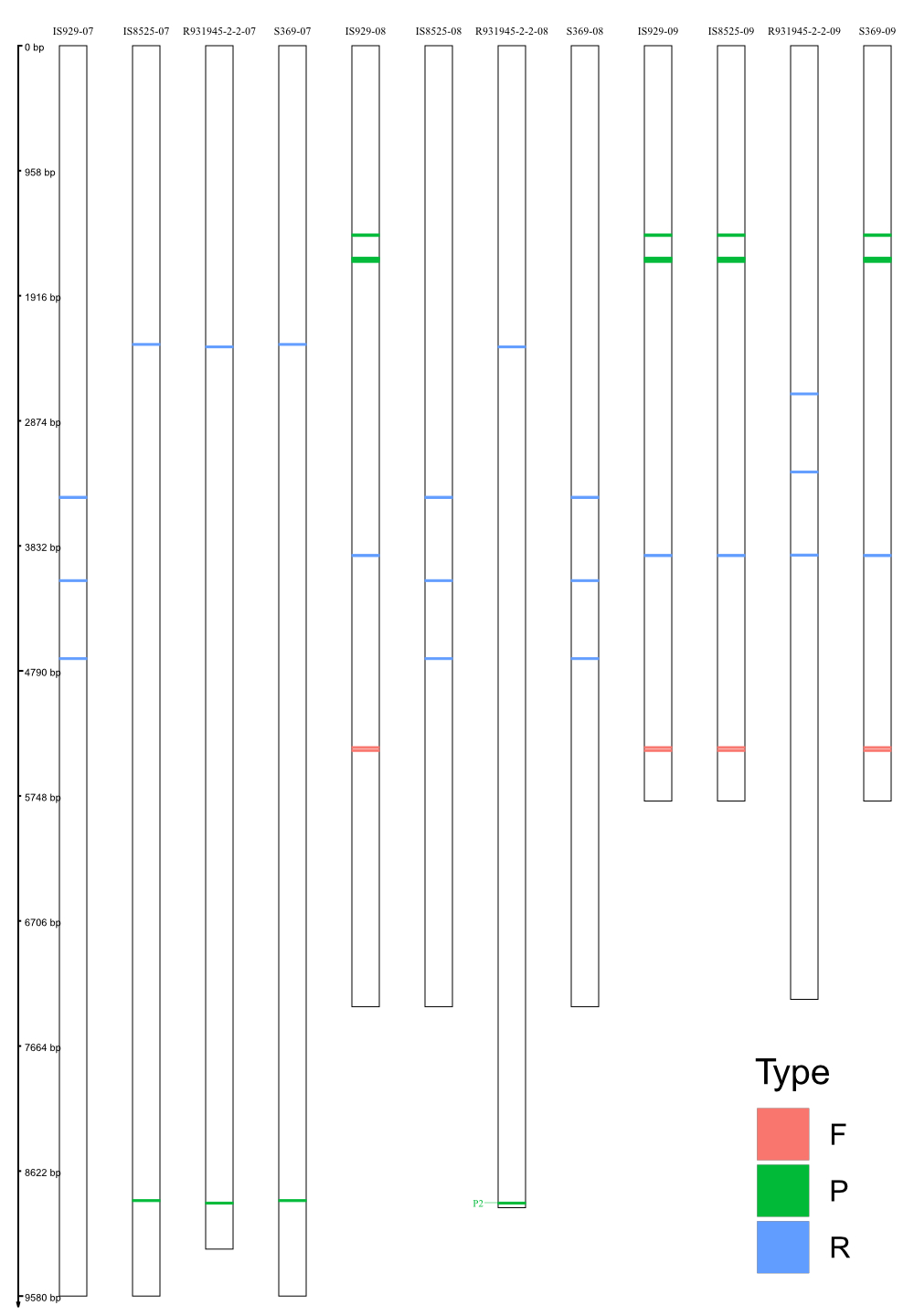


**Supplementary Fig. 12.** The location of forward repeats (F), reversed repeats(R) and P palindromic repeats (P) in seven sorghum plastid genomes
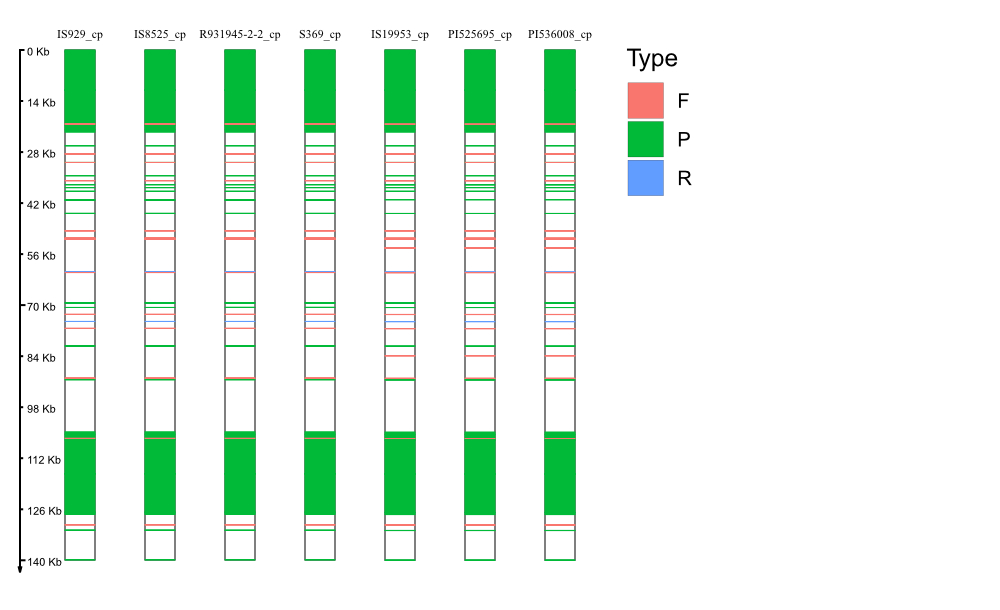
.


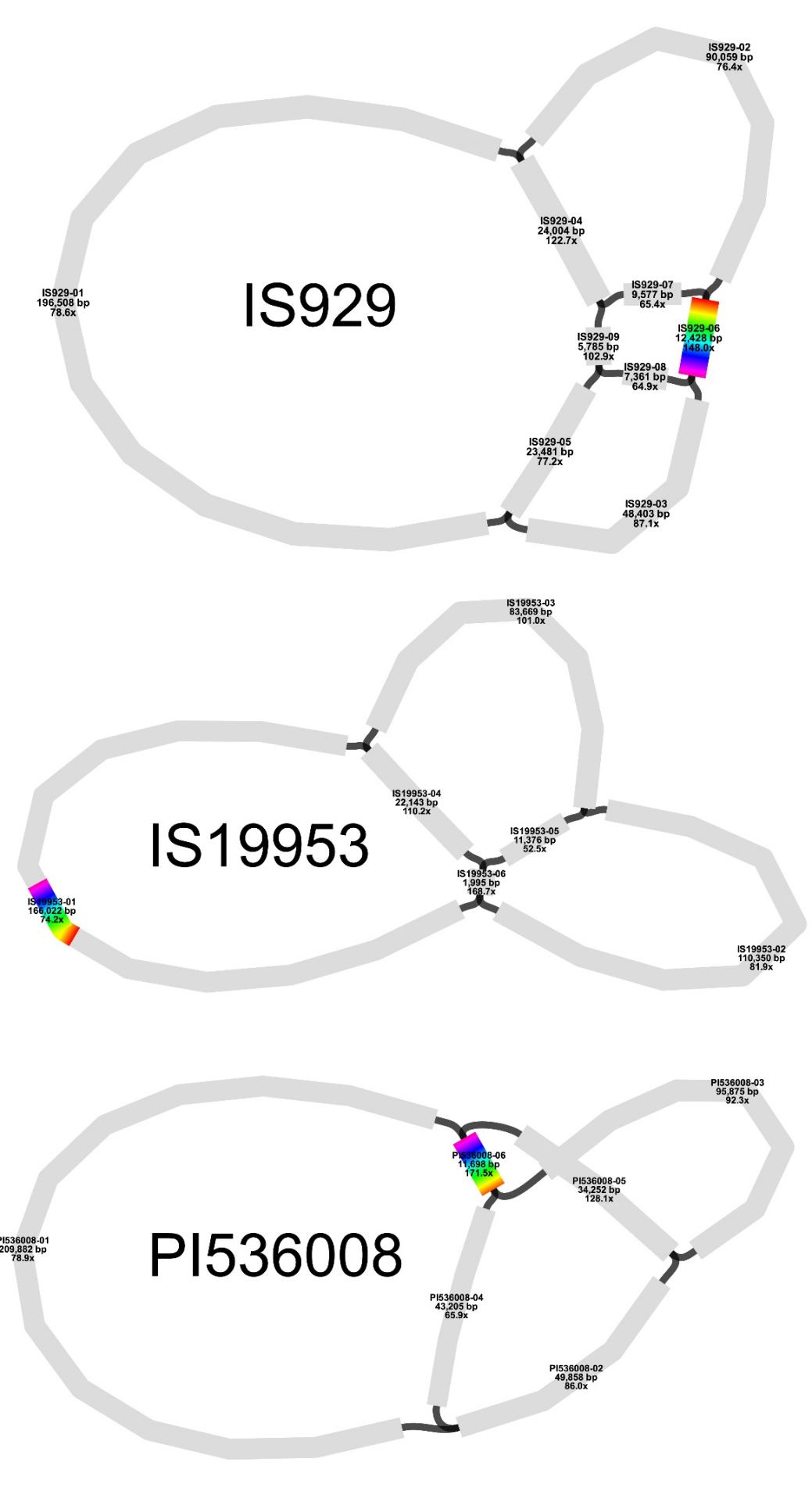


**Supplementary Fig. 13.** Location of a 12.4 kbp repeat sequence from sorghum reference genome NC_008360.1 in each of three structural types.


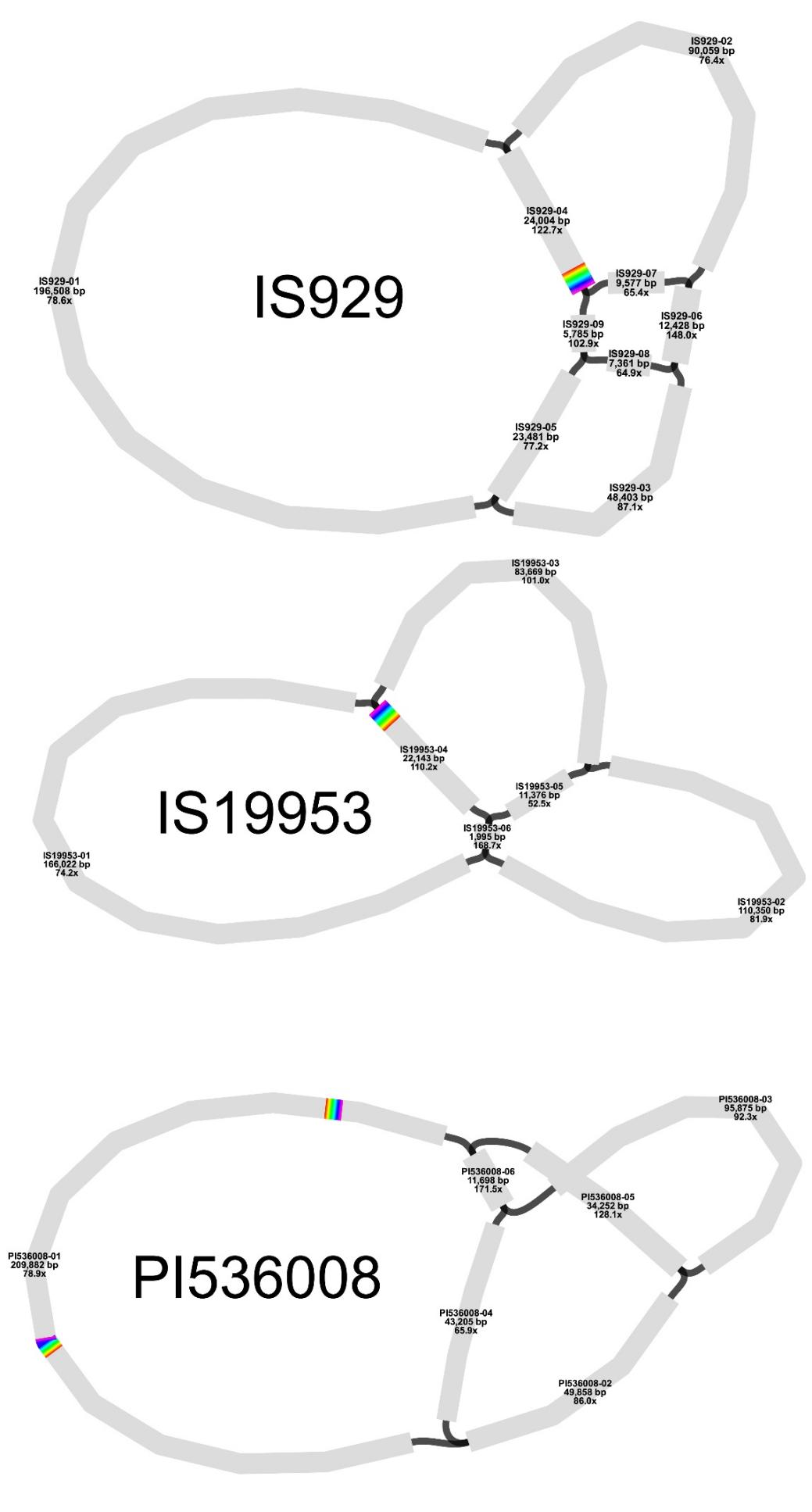


**Supplementary Fig. 14.** Location of a 3.6 kbp repeat sequence from sorghum reference genome NC_008360.1 in each of three structural types.


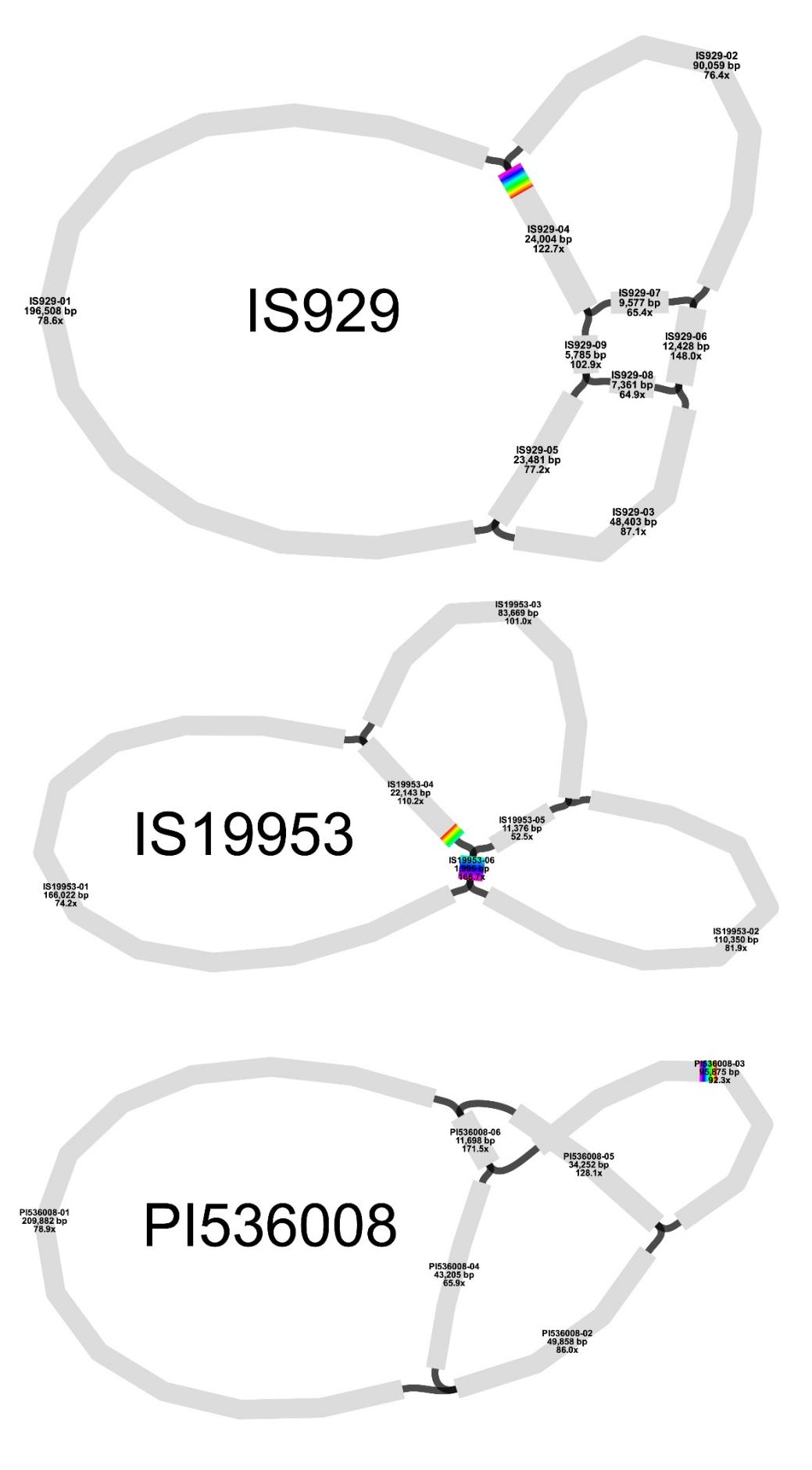


**Supplementary Fig. 15.** Location of a 4 kbp repeat sequence from sorghum reference genome NC_008360.1 in each of three structural types.


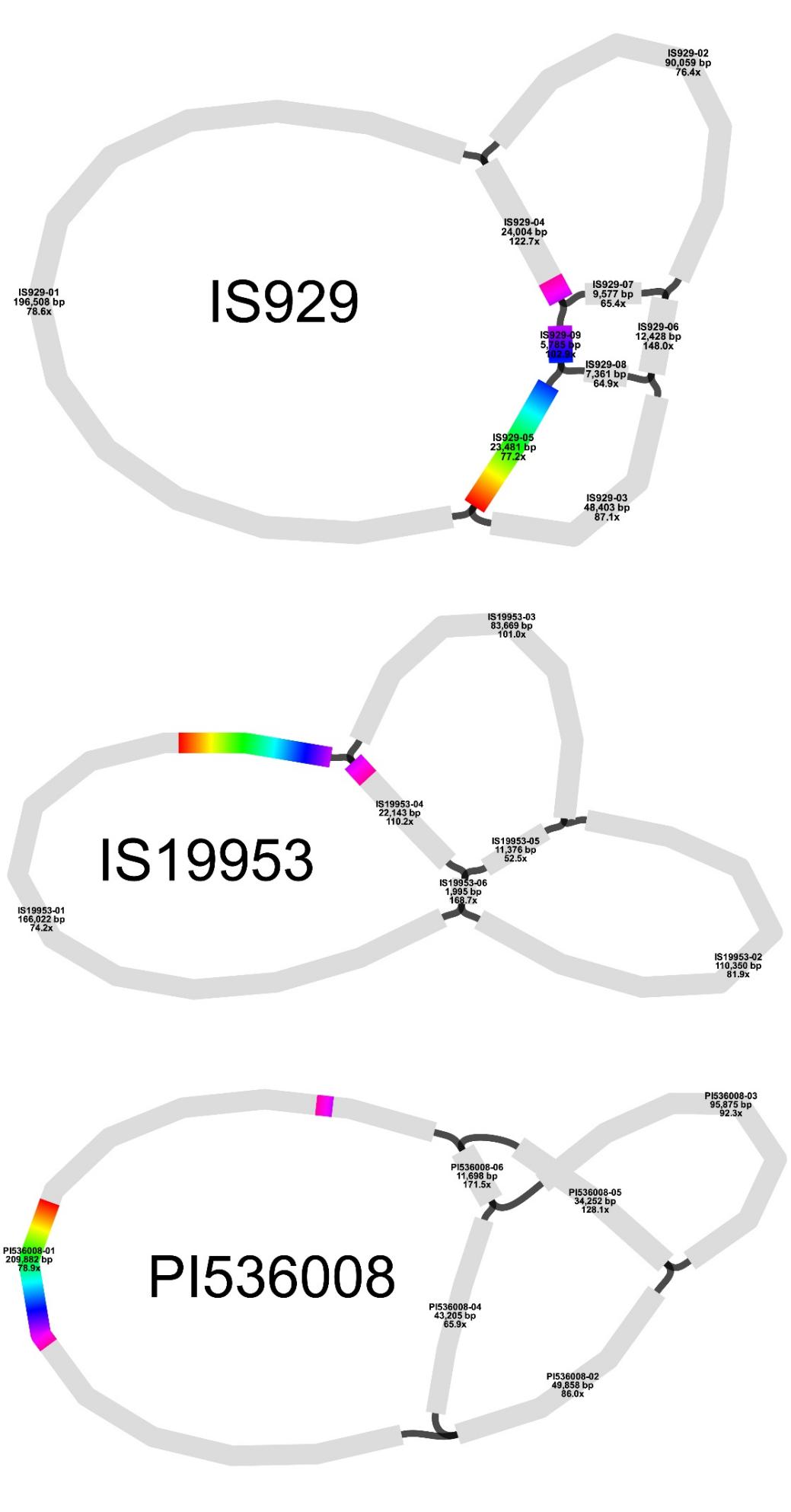


**Supplementary Fig. 16.** Location of a 33 kbp repeat sequence from sorghum reference genome NC_008360.1 in each of three structural types.


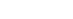

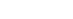


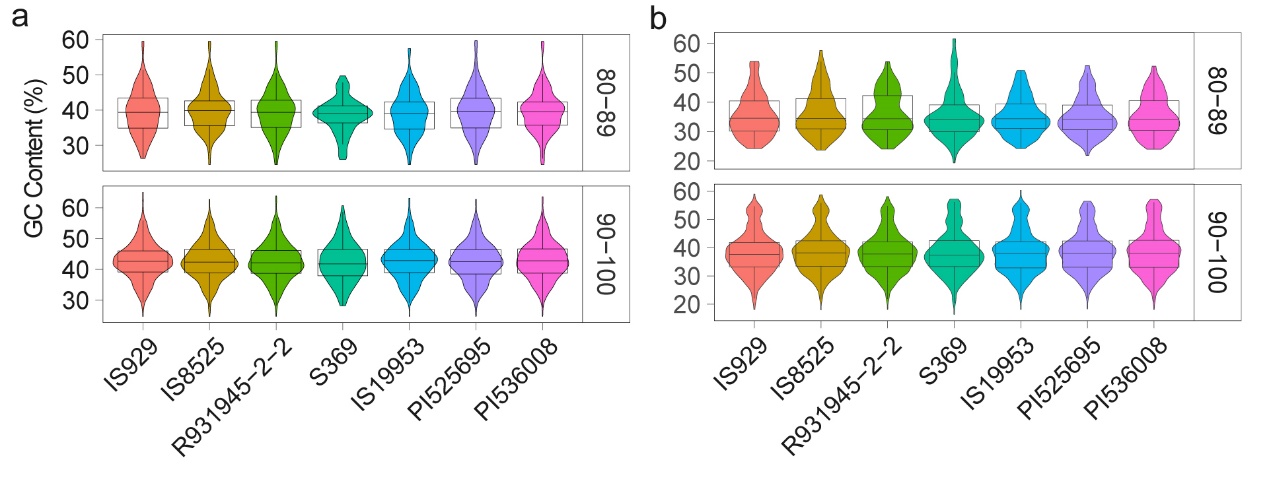


**Supplementary Fig. 17.** GC content of fragments transferred from sorghum organelles to the nucleus. a: GC content of NUMTs with different levels of sequence identity; b: GC content of NUPTs with different levels of sequence identity.


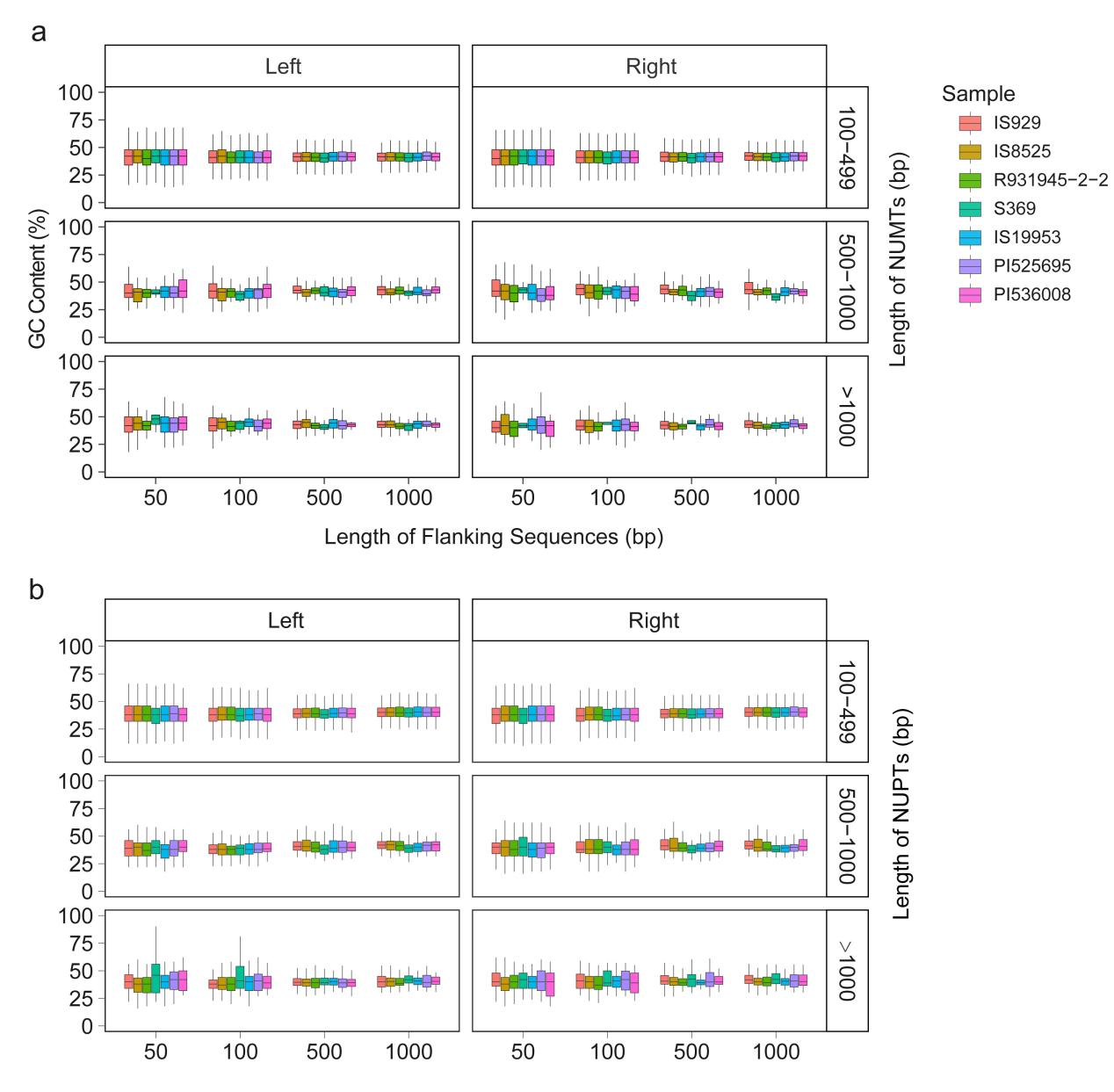
**Supplementary Fig. 18.** The GC content of transfer fragment flanking sequences a: The GC content of NUMT flanking sequences. b: The GC content of NUPT flanking sequences.


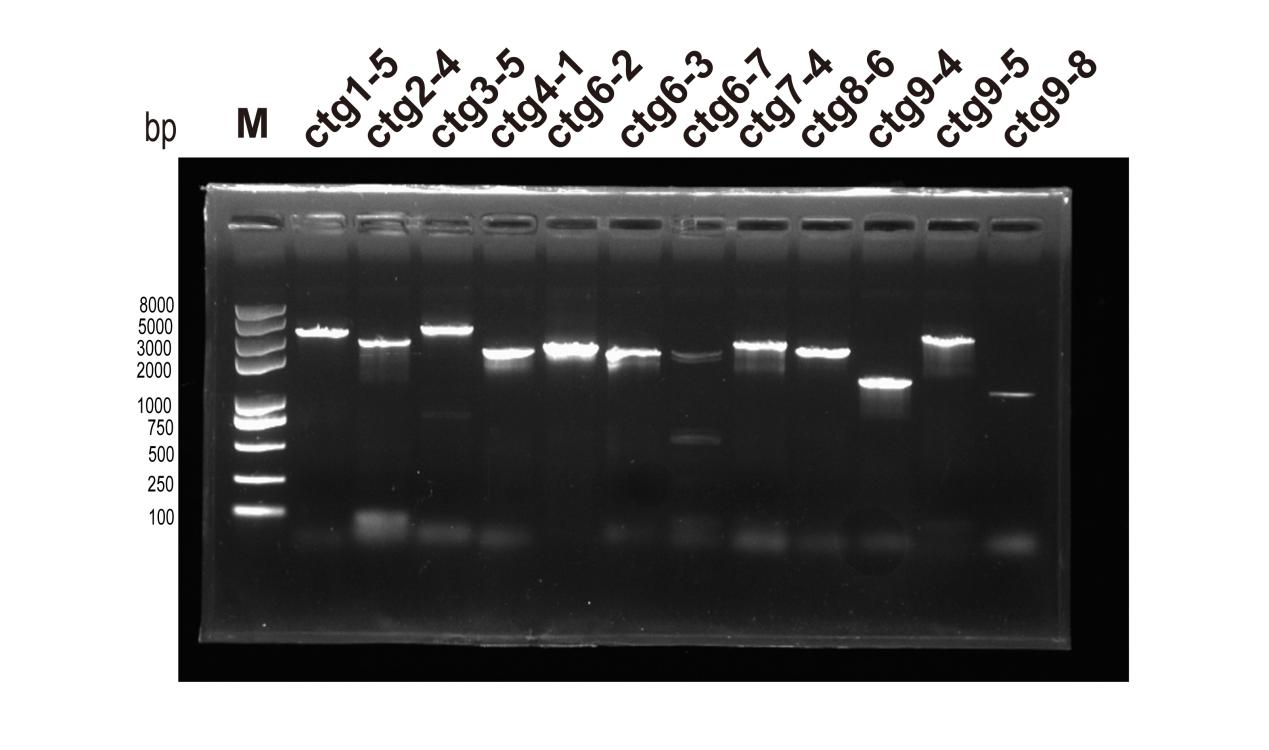


**Supplementary Fig. 19.** The full-length original gel of Figure 1d.
